# Empowering drug off-target discovery with metabolic and structural analysis

**DOI:** 10.1038/s41467-023-38859-x

**Published:** 2023-06-09

**Authors:** Sourav Chowdhury, Daniel C. Zielinski, Christopher Dalldorf, Joao V. Rodrigues, Bernhard O. Palsson, Eugene I. Shakhnovich

**Affiliations:** 1 Department of Chemistry and Chemical Biology, Harvard University, Cambridge, MA USA; 2 Department of Bioengineering, University of California, San Diego, La Jolla, CA USA; 3 Department of Pediatrics, University of California, San Diego, La Jolla, CA USA; 4 Novo Nordisk Foundation Center for Biosustainability, Technical University of Denmark, Kemitorvet, Building 220, 2800 Kongens Lyngby, Denmark

**Keywords:** Metabolomics, Biochemical networks, Machine learning, Target identification

## Abstract

Elucidating intracellular drug targets is a difficult problem. While machine learning analysis of omics data has been a promising approach, going from large-scale trends to specific targets remains a challenge. Here, we develop a hierarchic workflow to focus on specific targets based on analysis of metabolomics data and growth rescue experiments. We deploy this framework to understand the intracellular molecular interactions of the multi-valent dihydrofolate reductase-targeting antibiotic compound CD15-3. We analyse global metabolomics data utilizing machine learning, metabolic modelling, and protein structural similarity to prioritize candidate drug targets. Overexpression and in vitro activity assays confirm one of the predicted candidates, HPPK (*folK*), as a CD15-3 off-target. This study demonstrates how established machine learning methods can be combined with mechanistic analyses to improve the resolution of drug target finding workflows for discovering off-targets of a metabolic inhibitor.

## Abstract (web summary)

The authors present a workflow integrating metabolic perturbations with protein structural analysis to identify drug off-targets, demonstrating how combining machine learning methods with mechanistic analyses can benefit off-target identification.

## Introduction

Pharmaceuticals are often used with incomplete knowledge of their intracellular drug-binding partners^1^. For example, while antibiotic molecules are designed to selectively inhibit essential bacterial proteins^2^, even conventional antibiotic drugs are found to target multiple molecular targets inside bacterial cells. Identifying the full spectrum of drug targets is critical to understanding drug mechanisms of action as well as to exploit multivalency to tackle the problem of drug resistance.

Recent advances in systems biology tools are making systematic search for intracellular drug targets an increasingly accessible task^3^. Large-scale measurement of drug perturbations, such as metabolomics or transcriptomics, coupled to machine learning has been a promising approach to understanding drug mechanism of action and targeting. These methods can identify trends that are uniquely associated with a drug effect. However, identifying specific targets from machine learning is challenging due to the difficulty in interpreting such models. By contrast, mechanistic models and targeted analyses have the advantage of greater interpretability but struggle to learn from large-scale datasets.

Previously, we developed a novel antibiotic, the compound CD15-3, designed to interact with wild-type DHFR (dihydrofolate reductase) and its trimethoprim (TMP) resistant mutants^4^. In our previous report we showed that CD15-3 interacts with DHFR; however, overexpression of the DHFR-encoding gene folA was only able to partially rescue CD15-3-induced growth inhibition. The lack of complete growth rescue from overexpression of DHFR indicated the presence of an additional non-DHFR intracellular target of CD15-3, which could be responsible for the growth inhibitory effect of the compound^4,5^.

In this work, we develop a multiscale drug target-finding workflow that integrates machine-learning analysis of metabolomics data with metabolic modelling and protein structural analysis. We apply this workflow to search for the off-target of CD15-3. First, we analyse untargeted global metabolomics with statistics and machine learning to unravel differences in the global metabolome upon antibiotic treatment and captured potential hotspots of metabolic perturbation. We then integrate metabolic supplementation growth rescue experiments with metabolic modelling to identify metabolic pathways whose inhibition is consistent with data. Finally, we perform protein structural analysis to identify likely targets within candidate pathways based on similarity to known targets and validate these candidates experimentally using a combination of gene overexpression, imaging experiments and in vitro enzyme assays.

## Results

### An integrated metabolomics-guided framework to identify intracellular drug targets

**Fig. 1:**
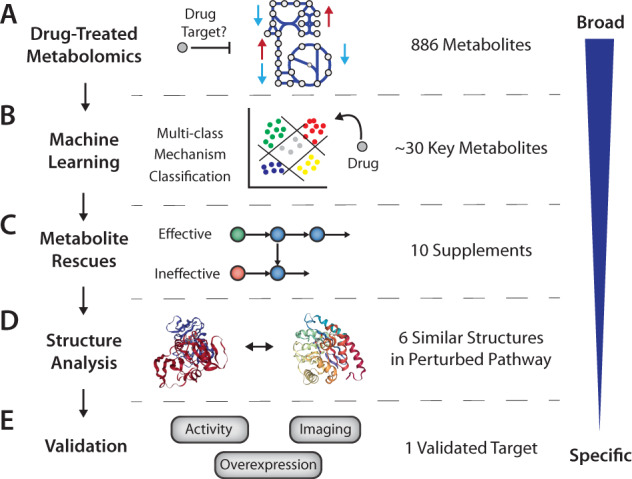
Metabolomics-guided drug target finding workflow. **A** Cells are treated with the compound of interest, and metabolite changes are measured with high throughput metabolomics. **B** Machine learning is used to identify drug-specific perturbations by comparison to publicly available drug response metabolomic profiles. **C** Key perturbed metabolites are provided as supplements to attempt growth rescue, and metabolic modeling is used to analyze the results to determine whether patterns are consistent with inhibition of certain metabolic pathways by the compound. **D** Structural analysis identifies candidate enzymes through homology to known targets of the drug. **E** Candidate genes are validated through a combination of overexpression of the target to rescue growth, activity assays to verify binding and inhibition of the target, and imaging to determine phenotypic effect.

Antibiotic molecules often have unintended intracellular targets. A systems-wide analysis capturing the drug-induced intracellular perturbation can be insightful in unravelling the intracellular mechanism of action of the drug and its molecular target. We developed an untargeted global metabolomics-guided multi-layered analysis framework for antibiotic off-target identification to deploy to search for the unknown target of CD15-3 (Fig. 1).

This workflow analyzes drug-treated metabolomics data using a combination of machine learning, metabolic modeling, and protein structures to prioritize candidate targets of antimicrobial inhibition. First, we perform an untargeted metabolomic analysis to identify metabolites that are highly perturbed by the drug, obtaining a broad assessment of drug activity, as well as evaluating the ability of these key metabolites to rescue growth (Fig. 1A). Independently, we utilize machine learning and a previously published dataset of metabolomic response of diverse antibiotics^6^ to identify mechanism-specific and unique drug signatures in the metabolomic response (Fig. 1B). Then, we utilize metabolic modeling to identify metabolic pathways that when inhibited would result in measured patterns of growth rescue (Fig. 1C). Finally, we perform a structural analysis of global and active site properties to the intended target of the drug to identify likely off targets (Fig. 1D). Based on the evidence of these three analytical tools, we prioritized candidate targets for experimental validation using gene overexpression, enzyme assays, and cell imaging to discern phenotypic changes (Fig. 1E).

We deployed this framework to search for the intracellular mode of action of an evolution drug lead CD15-3^4^ and determine its non-DHFR target.

### Metabolomic analysis of CD15-3 perturbation

**Fig. 2:**
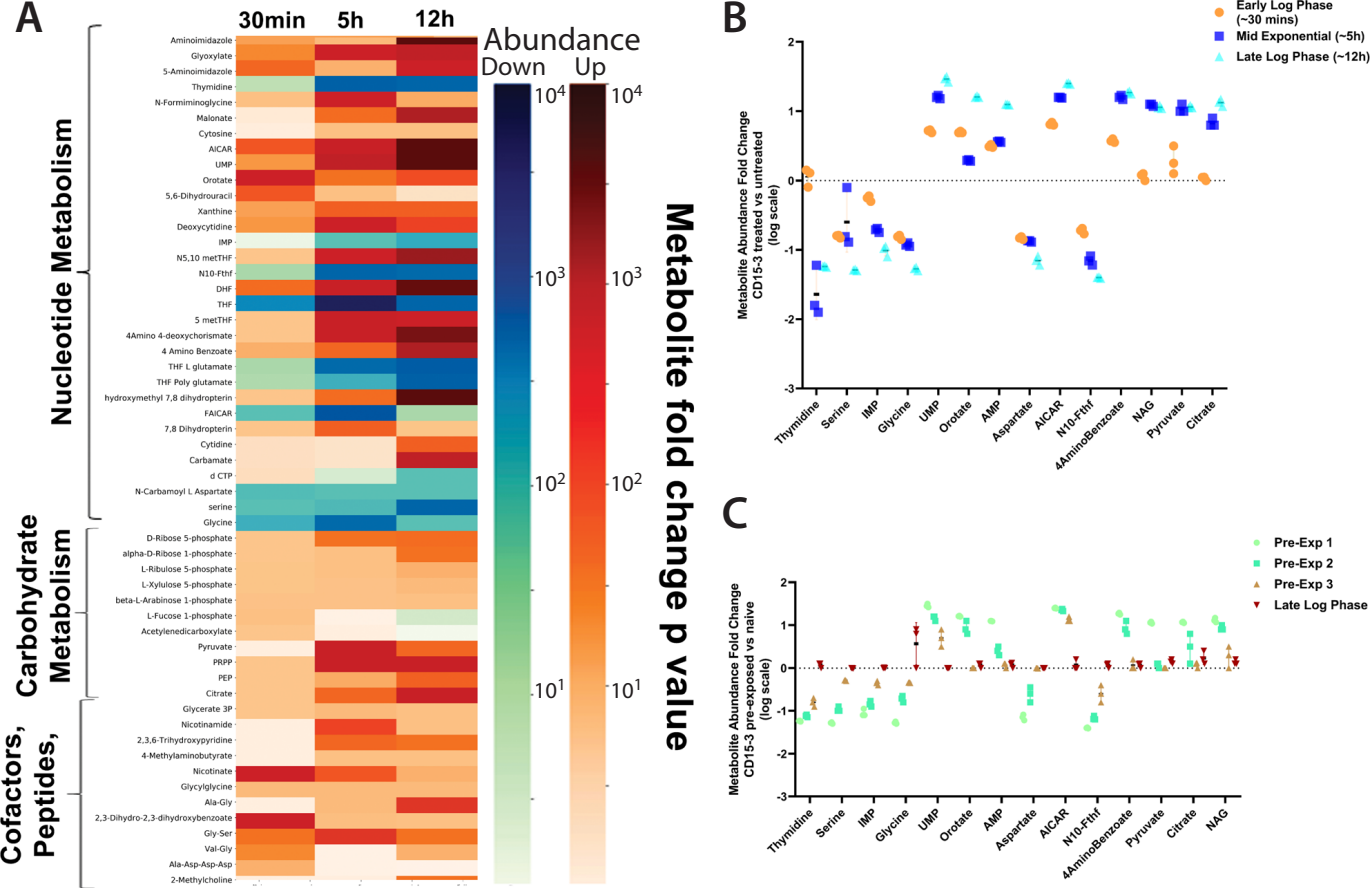
Metabolomics data following perturbation with the antibiotic candidate CD15-3. **A** Heat map describing the global metabolomic profile of untreated (WT [WT Escherichia coli BW25113]) and treated (WT + CD15-3) cells. Color gradient indicates fold differences (in log scale) observed in the metabolite abundance levels in the CD15-3 treated set compared to the untreated WT cells. Horizontal lines in the heat map indicate metabolites. Color bar shows negative logarithm of p-values (Mann-Whitney Test, one sided) associated with abundance ratios (treated vs. control) of each metabolite. A higher intensity of the blue bar indicates more depletion while a higher intensity of the orange bar indicates more accumulation. **B** Dot plot showing abundance fold differences (log scale) of selected metabolites as the cells grow in the presence of CD15-3. Metabolomics data for the treated and untreated control cells were gathered by harvesting cells at three different time points as shown in the figure. **C** Dot plot showing the metabolite abundance profile (log scale) of selected metabolites under the condition of the recovery experiment when cells pre-incubated with CD15-3 were grown in normal M9 media supplemented with 0.8 g/L glucose. The fold differences in the metabolite abundances are the ratios of metabolite abundance observed in the (recovering) pre-exposed/CD15-3 treated cells to that of the naïve cells. (* indicates metabolite abundance ratios with *p* values ≤ 0.05, ** ≤ 0.001, *** ≤ 0.0001 as derived from a one-sided Mann-Whitney test). n = 3 biologically independent samples were used for the experiments. All the data are presented as mean values +/- SEM. Source data are provided as a Source Data file.

We first measured the metabolic perturbation upon CD15-3 treatment to obtain a metabolome-wide view of CD15-3 action inside the cell. To that end we carried out untargeted global metabolomics measurements to obtain the comparative global metabolome under untreated and CD15-3 treated conditions (Fig. 2). Cells were grown in the presence and absence of CD15-3 for different lengths of time and were harvested for processing at their respective early lag phase, midexponential phase, and late log phase (Supplementary Fig. 1). Comparative metabolite abundances showed progressively increasing differences in cellular metabolism as the cells were exposed to CD15-3 for longer lengths of time. Figure 2A shows the differences in metabolite abundances involved in nucleotide metabolism, carbohydrate metabolism, cofactors, and peptides. As observed CD15-3 significantly impacts the global metabolism of cells in the mid-exponential phase, with even greater differences being observed in the late log phase.

Thymidine, a constituent of the pyrimidine biosynthesis pathway shows a 15-fold drop in abundance in the midexponential phase with CD15-3 treatment and a 17-fold drop in the late log phase (Fig. 2B). 4-aminobenzoate, which is synthesized from chorismate and is an important metabolic intermediate leading to the biosynthesis of a host of crucial metabolites such as folates, shows 15-fold higher abundance in midexponential phase and 18-fold higher abundance at 12 hours of growth with CD15-3 treatment. N10-formyl-THF, a precursor in the THF biosynthesis, showed 12-fold upregulation in the midexponential phase with CD15-3 treatment and 15-fold higher abundance in the late log phase. Folates are crucial for the biosynthesis of many important cellular metabolites, including glycine, methionine, formyl-methionine, thymidylate, pantothenate and purine nucleotides. Our comparative global metabolome showed significant fold differences in many of these metabolites indicating some plausible perturbation around the folate pathway and gross perturbation distributed throughout the overall nucleotide metabolism. For example, serine and glycine showed continuous cellular depletion upon CD15-3 treatment with more than 20-fold lower abundances at late log phase under CD15-3 treatment. Another interesting metabolic marker for perturbed purine metabolism is AICAR, which showed almost 8-fold higher abundance in the early lag phase ( ~ at 30 minutes of growth) and 16-fold higher abundance in midexponential phase. The cellular buildup of AICAR at early stages of treatment could indicate that purine metabolism gets disrupted quite early under CD15-3 treatment. UMP, a constituent of pyrimidine metabolism also showed cellular build up with 32-fold higher abundance at late log phase with treatment. Also, significant fold differences in the abundance levels of various peptides, cofactors and lipids were observed which too could be attributed to a CD15-3-induced metabolic stress response^4^. We observed significant fold differences in some metabolites constituting carbohydrate metabolism. Pyruvate and citrate cellular buildup has been known to be associated with metabolic stress response^7^. Under CD15-3 treatment we observed 11-fold higher abundance of pyruvate and 12-fold higher abundance of citrate at the late log phase of growth with CD15-3 treatment. Cellular buildup of citrate under CD15-3 treatment potentially indicates a possible slowdown of glycolysis and in turn energy metabolism.

We went on to determine the abundance levels of the relevant metabolites during recovery following CD15-3 treatment, with the hypothesis that the metabolites displaying delayed recovery after CD15-3 treatment may be more impacted by the treatment. We incubated WT cells (WT Escherichia coli BW25113) in CD15-3 for 12 hours and subsequently transferred them to M9 media supplemented with 0.8 g/L glucose. In a parallel control set, WT cells were grown for 12 hours in M9 medium with glucose at 0.8 g/L and subsequently regrown in the same media. Metabolite abundance levels were measured at four discrete time points (Supplementary Fig. 2) of cell harvesting. Notably, while recovery dynamics of particular metabolites are likely impacted by the normal metabolic rate of pathways involving those metabolites, we saw that there was a diversity in recovery times across both low and high-activity pathways. AICAR and several other metabolites that showed significant abundance changes in early stages of treatment gradually restored to normal levels during recovery (Fig. 2C). However, thymidine, IMP, and serine had significant fold differences until the last pre-exponential phase (which we term pre-exponential phase 3 as shown in Supplementary Fig. 2 as PE3), which occurred between 3 and 5 hours. A similar trend was observed with AICAR, UMP, and N10-formyl-THF, as these metabolites took longer to recover. N-acetylglutamate (NAG), which is a constituent of the ornithine biosynthesis pathway via the formation of N-acetyl ornithine, had significantly higher abundance upon treatment and was found to respond much earlier in the recovery experiment with abundance levels quickly returning to normal.

### Machine learning reveals antibiotic mechanism-specific perturbations

**Fig. 3:**
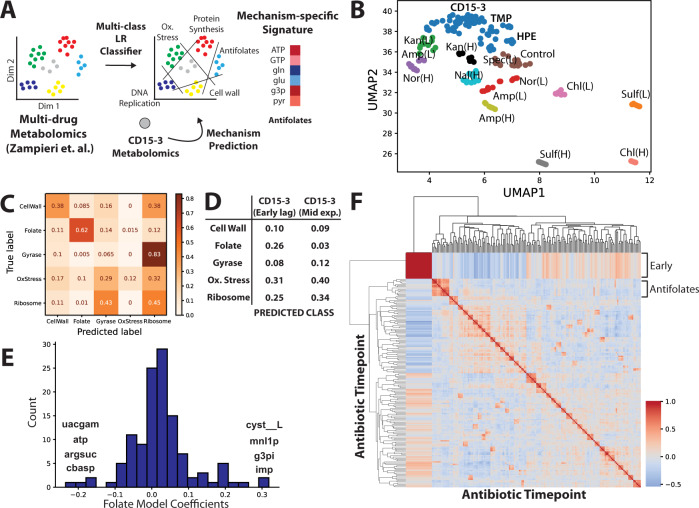
Machine learning identifies mechanism-specific trends in metabolomics data. **A** A multiclass Logistic Regression model was used to identify signatures associated with 5 different drug classes from a previous study (Zampieri et.al. 2017) This signature is removed from the CD15-3 metabolomics data to calculate a residual drug-specific signature that is examined for signs of additional targets. **B** Uniform Manifold Approximation and Projection (UMAP) plot of metabolomics data with clusters (*N* = 21) generated by Density-based spatial clustering of applications with noise (DBSCAN). **C** Confusion matrix demonstrates multi-class prediction accuracy for the antifolates class of antibiotics. Color scale shows prediction accuracy from 0.0 to 1.0. **D** Predicted drug mechanism, in the form of a probability p, from the logistic regression model for CD15-3 treatment at the early lag and mid exponential phase of growth. Values are rounded but columns sum to 1. **E** Model feature importance shows diverse metabolites contributing to the antifolate signature. **F** Correlation matrix of filtered metabolomics data shows that most drugs induce highly disparate perturbations as evidenced by lack of clustering behavior of samples, with exception to the antifolate class. Color scale shows correlation from −1.0 in blue to 1.0 in red.

To aid the interpretation of the measured metabolomics data, we developed a machine-learning workflow to identify metabolic signatures associated with both known and unknown targets of a compound. To contextualize the metabolomic response for CD15-3 and separate drug-specific effects from general growth inhibitory effects, we utilized a previously published survey of the metabolomic response of *E*. coli to diverse antibiotics^6^. We note that while a broad spectrum of previously analyzed drugs is desirable to help identify drug mechanism-specific metabolic responses, the compound of interest is not required to fall into one of these drug classes (Supplementary Data 1). We trained a multi-class logistic regression (LR) model to identify metabolomic perturbations associated with each of five possible mechanisms (antifolate, cell membrane, DNA synthesis, translation, oxidative stress) (Fig. 3A). Visualizing the data with Uniform Manifold Approximation and Projection (UMAP) and clustering the projection revealed that CD15-3 perturbation showed similarity to several other antibiotics including the DHFR-targeting antibiotic trimethoprim, as well as hydrogen peroxide perturbation that was used as a control to approximate a generic antibiotic growth inhibition (Fig. 3B). The multi-class LR model performed well for antifolates (Fig. 3C), the class of greatest interest, although it performed poorly for many other antibiotic classes. Consistent with the UMAP projection, the LR model suggests that while CD15-3 shows characteristics as an antifolate at early time points, it shows a broader growth inhibitory response consistent with non-specific antibiotic perturbation (Fig. 3D).

We further evaluated this model to identify metabolites that were key to distinguishing the antifolate response (Fig. 3E). While metabolite scores contributing to the antifolate class prediction were diverse, top hits included several purine and pyrimidine adjacent metabolites (ATP, IMP, Argininosuccinate, Carbamoyl-L-aspartate) consistent with antifolate inhibition of nucleotide metabolism. Among these, ATP and IMP were identified as perturbed from the statistical analysis of CD15-3 response, and these were utilized as targeted for growth supplementation experiments in the next section.

Investigating markedly better performance of the antifolate model over other classes of antibiotics, we found that the correlation of metabolomic response is substantially greater across different antifolates compared with other classes of drugs (Fig. 3F). The consistent perturbation seen in antifolates is logical given the critical metabolic role of the folate pathway in growth.

### Metabolic modeling predicts patterns in growth rescue experiments for candidate pathway inhibitions

**Fig. 4:**
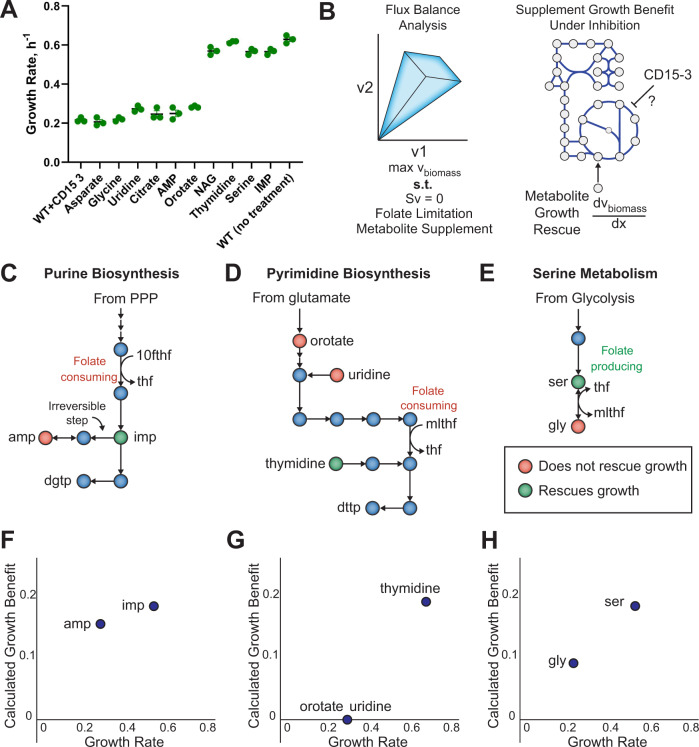
Effect of nutrient supplements on CD15-3-treated cells indicate broad inhibition of folate pathway. **A** Bar plot showing how metabolite supplementation impacts growth rate in the presence of CD15-3. Rescue from CD15-3-induced growth inhibition is observed with thymidine, serine, NAG and IMP supplementations. A fixed concentration of 200 µM CD15-3 was used in these supplementation experiments. *n* = 3 biologically independent samples were used for the experiments. Data are presented as mean values +/- SEM. Source data are provided as a Source Data file. **B** A metabolic supplementation strategy was employed for target identification by utilizing metabolic pathway analysis to find supplementation patterns consistent with inhibition of a particular metabolic pathway. Flux balance analysis was used to identify metabolic pathways with inhibition consistent with observed growth rescue patterns. **C** Purine biosynthesis pathway. Both IMP and AMP supplements are downstream of folate-requiring steps. However, AMP is unable to be converted to dGTP due to an irreversible step in the pathway, while IMP can be converted to both dATP and dGTP. **D** Pyrimidine biosynthesis pathway. The growth-rescuing supplement, thymidine, enters the pathway downstream of the folate-requiring step. Supplements that enter upstream of the folate-requiring step, namely orotate and uridine, did not rescue growth. **E** Serine and glycine biosynthesis. Serine, which produces the charged folate form 5,10-methylene tetrahydrofolate when converted to glycine, rescued growth, while glycine itself did not rescue growth. **F** Correlation of growth rate from different supplements with purine biosynthesis metabolites and corresponding model calculated growth benefit following a constraint on the folate-dependent reaction AICART in purine biosynthesis**. G** Correlation of growth rate from different supplements with pyrimidine biosynthesis metabolites and corresponding model calculated growth benefit following a constraint on the folate-dependent reaction TMDS in pyrimidine biosynthesis**. H** Correlation of growth rate from different supplements with serine metabolism metabolites and corresponding model calculated growth benefit in the form of folate cofactor regeneration.

The metabolite response to CD15-3 identified major perturbations in the nucleotide metabolism. Further the statistical and machine learning analysis revealed that CD15-3 metabolomic perturbation has both antifolate and generic antibiotic response signatures. To further narrow down which perturbations are most directly linked to CD15-3-dependent growth inhibition, we chose to use a subset of the identified metabolic markers for metabolic supplementation experiments to attempt a rescue from CD15-3-induced growth inhibition (Fig. 4A). To this end, wild-type cells were grown in the presence of externally supplemented metabolites under conditions of CD15-3 treatment. These externally supplemented metabolites were selected based on a combination of three factors: 1) perturbation of their abundance levels in the comparative global metabolome (CD15-3 treated versus untreated), 2) proximity to the folate pathway, as DHFR was the intended target for CD15-3^4^, and 3) practical considerations such as availability of a transporter for the compound in *E. coli* and commercial availability of the substrate (Supplementary Data 1 for available transporters). The selection of compounds was based ultimately on qualitative criteria, as quantitative perturbations of diverse metabolites were not expected to correlate with their functional significance. Instead, we tried to achieve wide coverage of compounds that appeared to have links to CD15-3 effects, which we could analyze further using the metabolic analysis approach described below. We expect that alternate sets of supplemented compounds could be chosen that would provide similarly useful information.

In all these experiments, the external metabolite supplement concentration was kept at 0.5 mM. Control experiments were performed in the absence of CD15-3 to check for intrinsic toxicity of these external metabolite supplementations (Supplementary Fig. 3A). We observed that the metabolites selected for the metabolic supplementation experiments did not show toxic effect on the bacterial growth. Also, we checked the possibility that these compounds might serve as alternate carbon sources. To that end we grew cells without CD15-3 in M9 minimal media with each of these supplements (without glucose). The growth profile was compared with bacterial growth in M9 medium with 0.8 g/L glucose, which is the typical media composition used in the rest of the study. We observed that, apart from thymidine, no other metabolite served as an alternate carbon source (Supplementary Fig. 3B). In the presence of thymidine, cells did show slow growth with a very long lag phase compared with the glucose control. Further our control experiments suggested that metabolic supplementation did not result in changes in the pH of the media and affect the lag-time of the growth (Supplementary Fig. 3C and D)

Supplementation with thymidine, NAG, serine, and IMP showed growth rescue, as reflected in improved growth rates (Fig. 4B) under the conditions of CD15-3 treatment. On the other hand, negligible or no effect on the growth rates were observed with external supplementation of aspartate, glycine, uridine, citrate, orotate and AMP. Thymidine and other metabolites, which showed significant rescue of CD15-3 inhibition improved the growth rate to around 0.6 h^−1^, which is comparable to the growth rates observed in the WT (WT Escherichia coli BW25113) cells in the absence of CD15-3.

It is interesting to note that metabolites whose external supplementation rescued growth rates did not significantly affect the lag time (Supplementary Fig. 3C). Lag time in bacterial growth is a critical indicator of cellular adaptation in the ambient growth condition^8^. In our experimental condition a higher lag time in the presence of some external supplements and CD15-3 treatment reflects time of metabolic rewiring and adaptation in the supplement-enriched growing condition. It is interesting to note that NAG and serine significantly prolonged the lag time (Supplementary Fig. 3C) under treatment conditions although both have shown to have positive impact in improving growth rates (Fig. 4B). Thus, NAG and serine could be considered as partial rescuers from CD15-3 induced stress with improvement on only growth rate and worsening the lag time.

Metabolic supplementation demonstrated that diverse compounds were able to rescue inhibition by CD15-3, while other metabolites had no effect. To better understand the metabolic rationale for the supplementation rescue patterns of CD15-3, we utilized the most updated metabolic network reconstruction of *E. coli*, iML1515^9^. We examined the trends in metabolite supplementation rescue experiments with respect to their location in corresponding metabolic pathways. We observed that the effectiveness of the supplement in rescuing growth was determined by the position of the supplement in the metabolic network viz a viz folate metabolism. Specifically, supplements that have the potential to mitigate folate deficiency, namely thymidine, IMP, and serine, were effective at rescuing growth. Supplementation with Thymidine and IMP bypass the folate-dependent step in pyrimidine and purine biosynthesis, respectively (Fig. 4C, D), while serine can contribute to folate production through its conversion to glycine (Fig. 4E). Meanwhile, supplements that do not mitigate a folate deficiency do not rescue growth, even though these metabolites may be adjacent in the network to successful supplements, such as uridine, AMP, and glycine. Uridine is a pyrimidine precursor that is upstream of the folate-dependent biosynthetic step. Glycine has an unclear role, as metabolism through the glycine cleavage chain produces folate, while conversion to serine consumes folate. Thus, although DHFR may not be the primary target of CD15-3, the inhibitory activity of CD15-3 still appears to primarily work through folate limitation.

To rigorously evaluate the hypothetical effect of these supplements, we developed a constraint-based metabolic modelling workflow to assess whether inhibition of a particular pathway is consistent with observed growth rescue patterns of supplements (Fig. 4B, Methods). Constraint-based modelling through flux balance analysis utilizes a metabolic network reconstruction to predict reaction flux through the metabolic network that maximizes growth for a given experimental condition^10^. We evaluated two possible metabolic inhibition scenarios: direct pathway inhibition, and cofactor depletion (see Methods). To evaluate possible direct pathway inhibition, we computationally inhibited metabolic reactions one by one and calculated the ability of each metabolite in the model to rescue growth. To evaluate possible cofactor depletion, we generated cofactor draining reactions and calculated the ability of each metabolite in the model to generate additional cofactor charges. Finally, we correlated the model-calculated benefit of each metabolite to the experimental observed growth rescue potential of those metabolites.

We found that the experimental growth rescue pattern was most consistent with a folate cofactor drain mechanism of CD15-3 (Fig. 3G). Comparing the model calculated to the observed growth benefit of different metabolites revealed an ability of the model to distinguish the benefit of similar metabolites. For example, the improved ability of IMP over AMP to rescue growth inhibition from the folate-dependent reaction AICART in purine biosynthesis (Fig. 4F), the improved ability of thymidine over uridine to rescue growth (Fig. 4G), and the ability of serine but not glycine to rescue growth (Fig. 4H), were all predicted correctly by the model, after accounting for wild type enzyme expression as detailed below.

We note that the metabolic model did not inherently incorporate the wild-type expression state under which supplements were administered. To correct for wild-type gene expression, we shut off flux through several reactions based on measured lack of expression under wild-type conditions^11^. For example, the model initially predicted citrate to have a growth-rescuing effect; however, the citrate transporter is not expressed under normal conditions. Similarly, the model calculated only a minor benefit of IMP over AMP initially, but investigation revealed that the AMP incorporation pathway utilized by the model included both spontaneous reactions, which are not likely to occur at a high enough rate to sustain growth, and the cryptic gene adeD, encoding adenine deaminase. These cases were handled individually utilizing available expression data^11^, and all model corrections are included in the metabolic modelling code in the Supplementary Data 1. We note, however, that this procedure can be automated through the use of context-specific metabolic modelling to account for gene expression^12^ or through the use of gene-protein-reaction relationships to identify and shut off potentially problematic spontaneous reactions.

To determine whether the observed agreement was specific to folate inhibition or was associated with growth more broadly, we additionally implemented random pathway inhibitions and compared the growth benefit under these conditions. While some metabolites, such as serine and glycine, had growth rescue behaviour that agreed more broadly with general growth benefit, other metabolites such as AMP/IMP and thymidine/uridine agreed substantially better with folate inhibition than inhibition of other pathways (Supplementary Fig. 4). Thus, the metabolic modelling results were consistent with topological pathway analysis to point to folate inhibition as the key metabolic limitation induced by CD15-3, despite DHFR being ruled out as the sole growth limiting enzyme by previous work^4^.

### Structural analysis of possible alternate binding targets

**Fig. 5:**
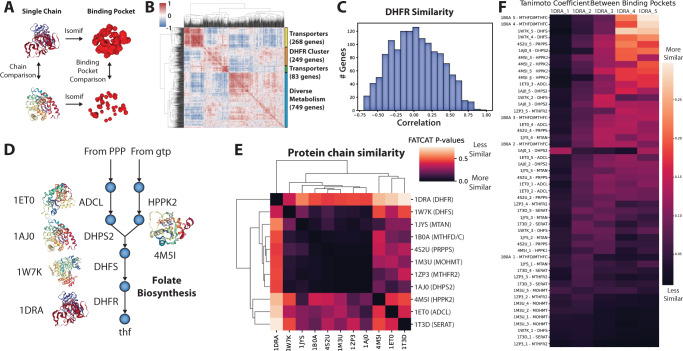
Protein structural similarity evidence supports other folate enzymes as possible targets of CD15-3. **A** Overview of workflow for structural analysis. **B** Global pairwise structural similarity (Pearson correlation R) across metabolic proteins based on calculated structural properties from the E. coli GEM-PRO. **C** Global structural similarity (Pearson correlation R) of DHFR, the intended target of CD15-3, to other genes. **D** Overview of folate pathway and protein structures extracted from PDB. **E** Results for whole structure similarity analysis utilizing FATCAT. Low *p*-values reported by FATCAT indicate more similar structures. **F** Results for active site analysis using isomif for comparison. Protein structure identifiers are taken from PDB. Color scale shows FATCAT *p*-values from 0.0 in black to 1.0 in white.

Experimental evidence from metabolomics supplementation, all suggested that folate perturbation is the primary mode of action of CD15-3. Thus, we hypothesized that the alternate target of CD15-3 also lies within the same metabolic pathway as DHFR. Notably, other enzymes in the folate biosynthetic pathway are also known to be drug targets, such as Dihydropteroate synthase, the target of sulfamethoxazole. To narrow down likely alternative targets of CD15-3, we employed a genome-scale reconstruction of enzyme protein structures, termed the GEM-PRO or Genome-scale Model with Protein Structures of *E. coli*^9^ (Fig. 5A). We first computed the overall similarity of global structural properties of all protein chains in the GEM-PRO, which showed DHFR and other folate pathway enzymes clustered separately from the majority of metabolic enzymes (Fig. 5B). We found that DHFR did not have a high degree of structural similarity to any particular enzyme based on whole chain property similarity (Fig. 5C). Utilizing the FATCAT algorithm for structural alignment^13^ and isomif analysis of annotated binding sites, we compared aligned structural similarity of the intended target DHFR and other enzymes in the folate biosynthetic pathway (Fig. 5D). While similarity of whole chains was generally low (Fig. 5E), comparison of active sites suggested possible alternative binding targets for CD15-3 (Fig. 5F). Notable proteins with high binding pocket similarity scores included several enzymes involved in folate biosynthesis and folate interconversion, such as MTHFC, HPPK2, and DHPS. Thus, we decided to screen several candidate upstream enzymes in the folate biosynthetic pathway whose binding pockets have high similarity to DHFR.

### In vivo validation of the intracellular target of CD15-3

Utilizing the prioritized target list from structural analysis, we evaluated whether overexpression of any of these enzymes rescued growth inhibition by CD15-3. Regulated overexpression of candidate proteins viz., HPPK (encoded by gene *folK*), DHPS (encoded by gene folP), DHFS (encoded by folC), MTHFC (encoded by gene folD), MTHFR (encoded by gene metF) and ADCL (encoded by gene pabC) using the pBAD promoter with 0.1% arabinose induction was carried out to determine whether any of the overexpressed genes show recovery from CD15-3 induced growth inhibition and thus may be the non-DHFR target of CD15-3. In our previous study^4^ we showed that overexpression of folA (encoding DHFR) partially rescued CD15-3-induced toxicity at lower concentrations of CD15-3.

**Fig. 6:**
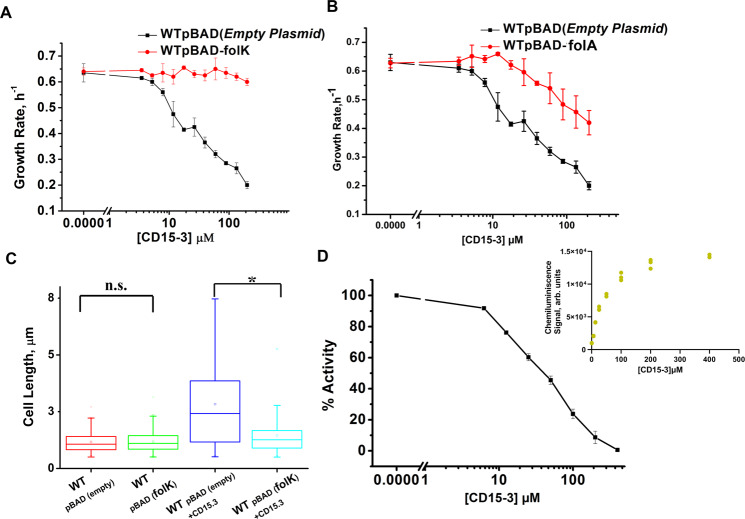
Overexpression of folK (encoding HPPK) rescued cells from CD15-3 induced growth inhibition. **A** Overexpression was induced with 0.1% arabinose and folK was expressed under pBAD promoter. *folK* rescued growth rate at all CD15-3 concentrations. **B** Overexpression of *folA* (encoding DHFR) was induced at 0.005% arabinose. *folA* overexpression had only partial effect in recovering growth rate in CD15-3 treated cells. Growth rates for control (pBAD empty plasmids) and each of the conditions tested (*folk* and *folA* overexpressions) were derived from at three biologically independent bacterial (WT Escherichia coli BW25113) cell cultures. **C** Distribution of cell lengths as derived from DIC imaging of WT (WT Escherichia coli BW25113) and WT cells overexpressing *folK* under control and CD15-3 treatment conditions. Untreated WT and cells overexpressing *folK* had comparable cell lengths with median cell lengths of 1.06 µm. (n.s. indicates the distribution of cell lengths were not significantly different, one-sided Mann-Whitney test, *p*-value = 0.952). Both WT and cells overexpressing *folK* were subjected to CD15-3 treatment at IC_50_ concentrations. WT treated cells had a filamentous morphology and the median cell length (2.41 µm) double of that of the untreated WT set. WT cells overexpressing *folK* after CD15-3 treatment had a median cell length of 1.252 µm which is slightly higher than that of untreated set. However, the cell size distribution of the cells overexpressing *folK* showed much less change after CD15-3 treatment compared to that observed for the WT (* indicates the distribution of cell lengths were significantly different, one-sided Mann-Whitney test, *p* value < 0.001). WT cells with empty p-BAD plasmid under no treatment had a minimum length of 0.5 µm and a maximum length of 2.27 µm. WT cells with p-BAD plasmid expressing *folK* under no treatment had a minimum length of 0.5 µm and a maximum length of 2.31 µm. WT cells with empty p-BAD plasmid under CD15-3 treatment had a minimum length of 0.5 µm and a maximum length of 7.48 µm. WT cells with p-BAD plasmid expressing *folK* under CD15-3 treatment had a minimum length of 0.5 µm and a maximum length of 2.78 µm. Imaging experiments were carried out for 4 times for each of the test conditions as well as the control. The statistics for cell length was derived after measuring cell length for >300 cells (300 to 400 cells) for each of the control and experimental conditions. The central band in the boxplots represents the median of the distribution, square-points inside the box represent the mean cell length of the distribution, the box ends represent the 25^th^ and 75^th^ percentiles, and the whiskers represent the 10^th^ and 90^th^ percentiles. **D** Inhibition of purified HPPK (encoded by *folK*) by CD15-3 in an in vitro assay (see text and Methods for details). Residual activity of HPPK at various concentrations of CD15-3 is shown. Inset shows the plot for the absolute chemiluminescence signals at various concentrations of CD15-3 in the HPPK assay buffer. At each of the CD15-3 concentration tested (from 0 to 400 µM) three individual chemiluminescence readouts were recorded. arb. units. in the inset figure refers to arbitrary unit. Activity was calculated from the chemiluminescence signals derived from three biologically independent repeats. All the data are presented as mean values +/- SEM. Source data are provided as a Source Data file.

Of the assessed proteins only overexpression of *folK* showed clear sign of rescue from growth inhibition (Fig. 6A). *folK* encodes for 6-Hydroxymethyl-7,8-dihydropterin pyrophosphokinase (HPPK). HPPK is a key enzyme in the folate biosynthesis pathway catalyzing the pyrophosphoryl transfer from ATP to 6-hydroxymethyl-7,8-dihydropterin, is an attractive target for developing antimicrobial agents^14–16^. Upon folK overexpression cells did not show any change in growth rates in a broad range of concentrations of CD15-3 (Fig. 6A). It stays close to 0.6 h^−1^ at all the concentrations of CD15-3, which is the typical growth rate of WT (WT Escherichia coli BW25113) in the absence of CD15-3. On the other hand, overexpression of *folA* at 0.005% arabinose induction showed only partial rescue from CD15-3 inhibition (Fig. 6B). Rescue in growth rate was more pronounced at lower CD15-3 concentration.

We further over-expressed *folP* (encoding DHPS) (Supplementary Fig. 5A), *folC* (encoding DHFS) (Supplementary Fig. 5B) and *folD* (encoding MTHFC) (Supplementary Fig. 5C) to see any possible promiscuous rescue effect on growth rates. Only at lower concentrations of CD15-3 (<50 µM) overexpression of these genes partially rescued growth inhibition. Overexpression of *folD* (Supplementary Fig. 4C) showed slight improvement in growth rate at midconcentration of CD15-3 (around 70 µM). *E. coli folD* gene encodes for a bifunctional enzyme having both methylenetetrahydrofolate dehydrogenase and methenyltetrahydrofolate cyclohydrolase activities. The dehydrogenase and cyclohydrolase reversibly catalyze oxidation of N5,N10-methylenetetrahydrofolate to N5,N10-methenyltetrahydrofolate and the hydrolysis of N5,N10-methenyltetrahydrofolate to N10-formyltetrahydrofolate and play critical role in purine metabolism.

As an additional negative control, we overexpressed two more genes and found no recovery effect. The *metF* gene encodes for methylene THF-reductase (MTHFR). Overexpression of *metF* did not show any rescue in CD15-3-induced growth inhibition (Supplementary Fig. 5D). Next, we overexpressed gene pabC, which encodes for ADCL. *pabC* encoding aminodeoxychorismate lyase is involved in the biosynthesis of p-aminobenzoate (PABA), which is a precursor of tetrahydrofolate. ADCL converts 4-amino-4-deoxychorismate into 4-aminobenzoate (PABA) and pyruvate. Overexpression of *pabC* did not show any recovery of growth rates in CD15-3 treated cells (Supplementary Fig. 5E). We performed rigid molecular-docking analyses and observed similar trends in the CD15-3 binding efficiency against the tested folate pathway proteins (Supplementary Table 1). Interestingly when similar over-expression strategy was deployed against the anti-folate compound Trimethoprim (TMP), overexpression-induced rescue-phenotype was not observed for any of the selected folate pathway genes. This could be attributed to the fact that TMP is designed specifically against WT DHFR and is a potent inhibitor^17^, (Supplementary Fig. 6A-F), while CD15-3 was designed to broadly neutralize (inhibit) WT and select DHFR escape variants^4^, thus leading to its interaction promiscuity

Of all the candidate genes, only *folK* overexpression showed a clear rescue effect at all studied CD15-3 concentrations, with growth approaching WT (WT Escherichia coli BW25113) level of around 0.6 hour^−1^ at all concentrations of CD15-3. This indicates strongly that CD15-3 interacts with cellular HPPK as its non-DHFR molecular target. The complete growth recovery observed with *folK* overexpression indicates that the growth-limiting folate perturbation originates in the *folK-mediated* step, which in turn impacts rest of the folate pathway. In the Supporting Text (Supporting Information) we provide an explanation as to why *folA* overexpression leads only to partial rescue while overexpression of *folK* resulted in full recovery from CD15-3 induced inhibition. In short, the reason for the difference is in different expression levels of *folA* and *folK* in overexpression experiment. The Supporting Text presented a quantitative analysis of the effects of the inhibition of two proteins by a common inhibitor and the competing factors of inhibition of both enzymes and sequestration of the inhibitor by an overexpressed enzyme. Supplementary Fig. 6 summarizes the effects of various metabolic supplements used and gene overexpression strategies deployed and show how CD15-3 has a folate-related mechanism of action.

Perturbation in the folate pathway leads to cellular filamentation and concomitant morphological changes^18–23^. WT cells treated and untreated with CD15-3 were grown for 4 hours and subjected to DIC imaging. CD15-3 treated cells (Supplementary Fig. 8B) showed a considerable extent of cellular filamentation as compared to untreated WT (Supplementary Fig. 8A) cells grown for the same length of time. A similar experiment was also done with WT cells overexpressing *folK* under pBAD promoter with 0.1% arabinose induction. WT cell sets overexpressing *folK* were grown for 4 hours under control and CD15-3 treatment conditions. WT cells overexpressing *folK* did not show any visible change in cellular shape and size (Supplementary Fig. 8D) compared to the untreated control (Supplementary Fig. 8C). Upon comparison of the median cell lengths (Fig. 6C), a slightly higher median cell length was observed in the *folK* overexpressing cells with CD15-3 treatment (median cell length = 1.252 µm), as compared to untreated cells under control conditions (median cell length = 1.06 µm). This slightly higher median cell length could be attributed to the fact that remaining CD15-3 unsequestered by HPPK also targets cellular DHFR. Overexpression of *folK* although mostly reverses the effects of CD15-3 on cell shape; The overall pronounced rescue in cell length upon overexpressing *folK* further supports the conclusion that HPPK is the non-DHFR cellular target of CD15-3.

### In vitro assay confirms CD15-3 is an inhibitor of HPPK encoded by *folK*

Next, we aimed to verify in an in vitro assay that CD15-3 indeed inhibits HPPK in a concentration dependent manner. To that end we performed a KinaseGlo^TM^ assay to test for HPPK activity and its probable inhibition in presence of CD15-3. We induced the overexpression of HPPK using IPTG and purified the protein for the activity assay (described in the methods section). KinaseGlo^TM^ assay is based on chemiluminescence^24^. With higher ATP concentration in the assay buffer, luciferase leads to the conversion of beetle luciferin to oxy-luciferin with the emission of light. The HPPK-mediated reaction utilizes ATP leading to ATP depletion and hence drop in the chemiluminescence signal. Any potential inhibition of HPPK would retain the original concentration of ATP keeping the chemiluminescence signal intact or like the control (with no HPPK activity). We observed a marked drop in the absolute chemiluminescence signal intensity in the HPPK reaction set with no CD15-3 in the assay buffer (Fig. 6D inset). Interestingly presence of CD15-3 led to enhanced absolute chemiluminescence signal intensity (Fig. 6D inset) suggesting that CD15-3 does inhibit HPPK. The inhibitory effect appeared to be progressively higher upon increase of the CD15-3 concentration in the reaction-assay-buffer. For control we performed similar experiments with 200 µM of CD15-3 with one ATP-dependent protein Adk, one ATP-independent protein BSA and three catalytically inactive mutants of HPPK (P43A, L45A and N55A) to validate that the drop in signal we observe in presence of CD15-3 in the HPPK reaction-assay set is due to the specific inhibitory interaction between CD15-3 and HPPK (Supplementary Fig. 9A). Catalytic sites were selected based on previously published works on pterin binding^15,25,26^. As expected, we did not observe any drop in luminescence with BSA, ADK and the three catalytically inactive HPPK mutants’ reaction set in the presence of CD15-3 suggesting that CD15-3 interacts specifically with WT (catalytically active) HPPK and inhibits its function, leaving unutilized ATP which contributes to the reported chemiluminescence.

In our assay protocol, HPPK reaction was initiated with the introduction of the substrate, 6-Hydroxymethyl-7,8-dihydropterin in the assay buffer (Supplementary Fig. 9A). We used the absolute chemiluminescence intensity values to calculate % activity using the following relation:

1 $$\%\,{Activity}=\frac{{Signal}\,{at}\,{inhibitor}\,{concentration}-{Signal}\,{at}\,{no}\,{reaction}}{{Signal}\,{at}\,{zero}\,{inhibitor}\,{concentration}-{signal}\,{at}\,{no}\,{reaction}}{{{{{\rm{X}}}}}}\,100$$

where signal at inhibitor concentration is the chemiluminescence signal at any given concentration of CD15-3, signal at no reaction is the optical signal obtained when the substrate is not added, and the reaction is not initiated.

Using this relation and plotting the absolute signal intensity values observed across the CD15-3 concentration gradient we found that HPPK retains 50% activity at 39.23 µM (IC_50_) (Fig. 6D) showing that CD15-3 is indeed an inhibitor of HPPK (encoded by gene folk). We further went on investigate if CD15-3 competes for the substrate binding site and carried out the chemiluminescence assay with an increasing concentration gradient of substrate (pterin) keeping CD15-3 concentrations fixed at 40 µM (~IC_50_). We observed that with an increasing substrate concentration CD15-3 induced inhibition is alleviated and chemiluminescence signal drops, suggesting the depletion of available ATP in the reaction mix (Supplementary Fig. 9B). This result suggests that CD15-3 competes with the substrate and binds at the HPPK active site. Derived from IC_50_ value obtained from the activity assay, we found that CD15-3 has an inhibition constant K_i_ of 3.54 µM.

## Discussion

Intracellular drug target identification is a hard problem. Often candidate drugs interact with unintended proteins inside cells and the resultant phenotypic effect emerges from the off-target protein(s). This applies to drugs spanning from antibiotics^1^ to anti-cancer formulations^27^. A systematic understanding of cellular targeting is critical in drug discovery programs as it provides mechanistic insights into intracellular drug action. This understanding in particular stands critical in the context of drug resistance, as drug-resistant cells can mount plethora of strategies to evade the drug action. For example, the bacterial “resistome” is a tight assembly of multi-layered highly orchestrated mechanisms^28^. In the current context of widespread antibiotic resistance, including the emergence of “multi-drug resistant” ESKAPE variants^29^, a mechanistic understanding of intracellular antibiotic targeting and what leads to bacterial death stands as an immensely pertinent problem.

Previously we reported CD15-3 as a potential antibiotic that significantly constrains bacterial evolvability by blocking the evolutionary escape routes which *E. coli* traverses under Trimethoprim selection^4^. We hypothesized that CD15-3 is an interesting lead towards the development of the evolution drugs to overcome antibiotic resistance. However, our in-cell experiments showed that CD15-3 has an additional unidentified non-DHFR target; thus, blocking more than one cellular target makes the evolution of antibiotic-resistant *E. coli* phenotypes more difficult. It was important to determine the alternative target of CD15-3 in *E. coli* cells to better understand the mechanism of action of this prototypic “evolution-drug” inside bacterial cells. In the present study we developed an integrated multiscale framework utilizing global metabolomics interpreted through machine learning and metabolic modelling, gene overexpression assays, and growth recovery studies eventually analysing data in the context of the metabolic network to unravel the unknown intracellular antibiotic target of CD15-3.

Antimetabolite classes of antibiotics such as antifolates (for example Trimethoprim) target proteins at critical points in the bacterial metabolic scheme. Hence an investigation of the metabolic architecture provides essential clues tracing the potential points of metabolic perturbations under conditions of antibiotic action. Critical analysis of such points of metabolic perturbation and its comparison with untreated control sets provides mechanistic insights of the drug action inside cell. Recent advances in untargeted metabolomics have provided valuable insights into the global metabolome and helped to quantitatively identify the metabolic cascades impacted by perturbations^6,30,31^. These datasets open the possibility of identifying the mechanisms of uncharacterized compounds through comparison to known profiles^32^. Also, machine learning methods have become increasingly popular for statistical analysis of the metabolomics data due to the inherent non-linear metabolomic data representation and the ability to process large and heterogeneous data rapidly^33,34^. While model interpretation has been a historical challenge in deploying machine learning for biological data analysis, interpretable ‘white box’ machine learning methods have come into focus as a viable area of development to empower drug discovery workflows^35^.

Employing machine learning on the comparative metabolomics data and training a multi-class K-nearest neighbour model, we found that CD15-3-induced metabolomic perturbation has a typical antifolate signature, suggesting that the unknown target is located somewhere in the folate pathway. Focusing on folate pathway perturbation and performing metabolic supplementation experiments we observed that supplementation with a subset of metabolites lead to growth recovery. Our analysis in the context of the metabolic network utilizing constraint-based metabolic modelling confirmed that inhibition of folate metabolism was consistent with patterns of growth rescue. We utilized protein structural analysis to suggest targets upstream of the DHFR catalysed step within the folate pathway and performed gene over-expression studies for these target-genes to determine which candidate targets rescue growth inhibition by CD15-3. Among all the candidate genes *folK*, which codes for HPPK, showed complete rescue of growth rate under CD15-3 treatment conditions. Unlike DHFR overexpression which only partially rescued CD15-3-induced growth inhibition^35^, HPPK showed clear recovery at all concentrations of CD15-3. In the Supplementary Note 1 we discuss a plausible explanation as to why we see a full rescue from CD15-3 induced growth inhibition with HPPK overexpression compared to partial rescue observed with DHFR overexpression.

The use of growth rescue experiments under various nutrient supplements was uniquely helpful in verifying the folate inhibition of CD15-3. We note that the choice of compounds to supplement in this study was based on a combination of identifying CD15-3 perturbed metabolites, metabolites close to the folate pathway, and metabolites that showed in in the machine learning analysis. We note that other metabolites could have been chosen that may have provided similar mechanistic information. For example, neighbouring compounds could be expected to provide similar information to the compounds we chose, provided that transporters are available for these compounds. Identifying metabolites ‘close’ to the folate pathway is a complex question as well due to the interconnected nature of the metabolic network. In the case of our study, we utilized a qualitative approach based on canonical pathway definitions established by manual curators, therefore matching a textbook understanding of proximity between metabolites.

This work demonstrates a promising path towards white box machine learning workflows through coupling standard machine learning modelling with mechanistic metabolic modelling and protein structural analysis. As the release of AlphaFold^36^ is making protein structure prediction an increasingly accessible task, this approach utilizing protein structural comparison could provide a new direction to rapidly demystify drug target identification (DTI). We note that in the case of CD15-3, we assumed a priori that the DHFR activity of the drug was important and could therefore analyse other likely targets based on similarity to this known enzyme target. For compounds with completely unknown targets, other approaches, such as docking and metabolite similarity assessment would need to be applied to generate an initial range of enzyme targets to analyse structurally. With no initial guess of where the initial target could be, a workflow still could be possible consisting of 1) metabolomics with comparison to previously characterized antibiotic compounds, 2) supplementation to probe growth inhibited pathways, and 3) overexpression of candidate targets to attempt a growth rescue. The structural analysis could still be deployed to computationally assess the possibility of multi-targeting of the drug.

With HPPK as the non-DHFR target, CD15-3 can be considered as a multivalent drug, which can simultaneously block and inhibit two molecular targets (DHFR and HPPK) inside the bacterial cell. Interestingly, being an essential protein, bacterial HPPK has been an attractive target for designing antibiotics^37^. Thus CD15-3 in principle can serve as a lead to a “monotherapy-analog” of “combination therapy” which blocks the emergence of antibiotic-resistant phenotypes by interacting with two targets making it difficult for bacteria to escape antibiotic stress. We note that, in principle, overexpression of any of the targets could sequester the drug, alleviating pathway inhibition as a resistance mechanism; however, this would come at a substantial proteomic cost itself.

We showed how integrating constraint-based metabolic modelling with machine learning in analysing large-scale metabolomics data can help capturing metabolic perturbation signatures and narrow down the search options for identifying intracellular drug off-target. We acknowledge that one limitation of the current proposed framework is this that it works with antimetabolite classes of antibiotics and has not been tested with antibiotics in general. Future work would aim at the development of a similar workflow for other classes of antibiotics where perturbations remain highly delocalized and impacts nonmetabolic cellular functions. Further, the problem of off-target activity is also highly relevant for drugs beyond antibiotics. Many anti-cancer drugs have been reported to have off-target toxicities^27^. We believe our proposed framework would be relevant in addressing these off-target identification problems. Future works would involve using this multi-scale framework for off-target identifications in other cellular models and complementing it with relevant functional assays.

## Methods

### Antibacterial Growth Measurements and IC_50_ values

Bacterial cultures of WT Escherichia coli BW25113 in M9 medium with glucose at 0.8 g/L were grown overnight at 37 °C and were then normalized to an OD of 0.1 using fresh medium. A new normalization to an OD = 0.1 was conducted after additional secondary growth for ~4 hours. Thereafter the M9 medium and six different concentrations of the CD15-3 in the 96-well plates (1/5 dilution) were incubated. The incubation of the plates was performed at 37 °C and the orbital shacking and absorbance measurements at 600 nm were taken every 30 min during 15 h. Growth rate was calculated using logistic fitting on matlab. The M9 media pH under the conditions of metabolic supplementation were continuously monitored throughout the culture-time.

### Metabolomics analysis

Untargeted global metabolomics was performed to understand the global metabolome of the WT (WT Escherichia coli BW25113) treated and control sets under different experimental contexts. In all the experiments cells were grown in M9 medium with glucose at 0.8 g/L in a 250 mL flask and temperature of 37 °C was maintained. Cells were pelleted with a brief 2 minutes precentrifugation incubation step on dry ice. After pelleting, the cell pellets were mixed with 80% pre-chilled methanol. Samples were thereafter vortexed and incubated in dry ice for 10 min followed by centrifugation at 4 °C for 10 min at maximum speed. The supernatant was collected, and the pellet was repeatedly processed by resorting to the above-mentioned procedures. Samples were stored at −80 °C until analyzed by mass spectrometry.

A Thermo q-Exactive Plus mass spectrometer coupled to a Thermo Ultimate 3000 HPLC was used to carry out the LC-MS analyses of metabolites in biological samples. The Electrospray source settings included a sheath gas flow rate and was set at 35, auxiliary gas flow rate at 5 L/min, a capillary temperature of 250 °C, and auxiliary gas temperature of 300 °C. Using the Thermo LC-MS Calibration mix immediately prior to the analysis calibration of the m/z range was performed. A scan range of 66.7–1000 m/z was used at a resolving power of 70,000 with alternating positive and negative ion mode scans. The chromatographic separation of metabolites was performed using hyrdophilic interaction liquid chromatography (HILIC) on a SeQuant ZIC-pHILIC column, 5 µm, polymer PEEK 150 mm×2.1 mm column (EMD Millipore) at a flow rate of 0.1 mL/min. Mobile phase A was 20 mM ammonium bicarbonate with 0.1% ammonium hydroxide, and mobile phase B was acetonitrile. The mobile phase composition was started at 100% B, and subsequently decreased to 40% B over 20 min. The column was then washed at 0% B for five minutes before re-equilibration to 100% B over fifteen minutes. The extracted ion currents were plotted using a mass accuracy window of 5 ppm around the predicted monoisotopic m/z value of the molecular ion of each metabolite. The integrated area of each peak was used to determine the response for each metabolite at their specific retention time as determined by chemical standards.

In all our experiments at least three independent biological replicates were analyzed. A list of 48 experimentally measured retention times was used for initial calibration of the retention time predictions. We performed data analysis for untargeted metabolomics using the software the packages MzMatch^38^ and IDEOM^39^. In untargeted analysis for peak assignment, we used IDEOM and included both positive and negative peak M/Z values and predicted retention times calculated based on chemical descriptors. We followed the same method of analysis as we had in one of our earlier studies^40^. For putatively identified metabolites the retention times were found to correlate fairly well with the values included in IDEOM (R^2^ = 0.7) and published in other studies (Pluskal et al., 2010) (R^2^ = 0.88 and R^2^ = 0.61, respectively). Based on this, additional metabolites from those sources closely matching IDEOM assignments were treated as standards in the identification routine. We also referred to KEGG and Ecocyc for additional chemical understanding of the metabolites.

### Recovery experiments

WT Escherichia coli BW25113 cells were grown both in absence and presence of prospective compound CD15-3 in M9 media supplemented with 0.8gL^−1^ glucose. Both the naïve (which were not exposed to CD15-3 treatment) and pre-exposed cells (cells treated with CD15-3) were subjected to growth in M9 medium with glucose at 0.8 g/L media and growth profiles were analyzed. The entire pre-exponential stage (lag phase) was grouped into equal time frames and both naïve and pre-exposed cells were harvested for metabolomic studies. Same process was also executed for harvesting both naïve and pre-exposed cells at late log phase.

### Overexpression experiments

WT Escherichia coli BW25113 cells were transformed with blank vector plasmids (without inserts) as well as plasmids overexpressing genes viz. *thyA, glyA, metF, purH, purC, folD, purD, adk* and *folK*. The genes were under pBAD-promoter and the overexpression of the genes was induced using externally supplemented arabinose (0.1%).

### Machine learning analysis of metabolomics data

Metabolomics data for ten antibiotics was taken from a published study^6^. The data was first filtered based on the criteria of having annotated, high confidence identities (annotation score > 50) and overlap (shared KEGG identifier) with the data generated in this study for CD15-3. Data was then averaged for each compound. Uniform Manifold Approximation and Projection (UMAP) was utilized to visualize the high-dimensional data in two dimensions, to inspect clustering behavior of the samples. A multi-class logistic regression model^41^ was trained to identify drug mechanism of action from metabolomics data. Five mechanisms of action were utilized from the original study: antifolates, cell wall synthesis, polymerase inhibition, translation inhibition, and oxidative stress. Mechanism labels were utilized as target values in a supervised learning approach. Zero time point data was excluded. UMAP and the LR algorithm were implemented using the scikit-learn Python package^41^. UMAP was implemented with 2 components, fixed random state of 42, and 14 neighbors, based on the number of samples for high and low concentration for a single drug. The LR algorithm was implemented with default hyperparameters, and performance was evaluated with leave-one-out cross-validation, utilizing different train-test splits and averaging performance of each run.

### Metabolic modeling analysis of metabolomics data

To analyze patterns in growth rescue data from metabolic supplementation, we utilized a flux balance analysis workflow^10^. We utilized MATLAB, the COBRA toolbox version 3.0^42^ and the latest metabolic network reconstruction of E. coli, termed iML1515^9^. Default options were used for the optimizeCbModel function.

To enforce folate limitation, growth was first optimized under glucose growth at an arbitrary but realistic uptake rate of 10 mmol/gdW/hr. Then, folate dependent reactions nearby to each metabolite supplement were identified. Reactions that consumed folate were limited to 90% of their optimal flux, and it was confirmed that growth rate was correspondingly limited. Then, metabolites were supplemented in silico at a rate of 0.1 mmol/gDW/hr. The calculated growth rate following supplementation was compared to the calculated growth rate before supplementation to determine the growth benefit of the metabolite under folate limitation.

Gene expression data was used to close flux through reactions that were not expressed under wild type conditions in a targeted manner around metabolites that were supplemented. Closing flux through all reactions that were not measured to not be expressed in the proteomics data was not possible due to lack of growth in the resulting simulations.

The resulting scores were combined for different pathway specific inhibitions for each supplement, as detailed in the code available in the Supplementary Data 1.

For non-folate pathway comparisons, reactions were constrained to 0 flux, and the benefit of each metabolite supplement for each reaction inhibition was calculated.

### Protein structural comparison

For the global structural analysis, general protein structural properties were taken from the *E. coli* GEM-PRO for all available metabolic enzymes. These properties were calculated previously as part of the GEM-PRO development. These structural data was standard normalized and then correlated with Pearson correlation to examine global structural similarity.

In the targeted folate pathway structural analysis, proteins were selected that were nearby in metabolic maps in the pathway of interest, as well as some unrelated metabolic proteins as a control comparison. Isomif ^43^ was used to pull different binding clefts from each of the selected proteins and the 5 largest of each protein binding clefts were kept. The similarity (Tanimoto coefficient) between the clefts of the *folA* protein structure (PDB ID: 1DRA) and each of the selected clefts of the proteins using isomif which was then plotted to view which binding clefts were most similar. FATCAT^13^ was used to compare the overall structural similarity between all of the selected proteins and the results were plotted using a clustermap to look for similar proteins. Visualizations of the proteins were created using nglview.

### Statistical and python plots

Statistical analyses of the data and their representation was carried out using Origin pro 8.1 package. Metabolomics data were processed using R based MS converting operation and IDEOM tool. For calibrating M/Z (mass to charge) values and retention times of the standard metabolites, XCalibur package was used. For the quantitative depictions of the metabolomics data statistically validated outputs were plotted using Python libraries of matplotlib and seaborn.

### Molecular docking

Using the AutoDock Vina 1.5.6 program, we performed the molecular docking study. We used the CD15-3 molecule in sdf format and converted it into pdb format using OpenBabel program^44^. We then selected the rotatable bonds in the ligand and prepared pdbqt file of the ligand molecule using Autdock Tool. Then we modified the candidate protein by adding polar hydrogen and calculated Kollman charges. We performed this docking analysis with exhaustiveness of 28, number modes of 10 and energy range of 4 by considering the candidate protein as rigid, whereas the ligand was flexible in nature.

### HPPK purification

Recombinant HPPK was over-expressed in *E. coli* (BL21 DE3 strain) after transforming the cells with pET 28 A plasmid which was obtained from Genescript. We induced the over-expression of HPPK with 1 M isopropyl-1-thio-*β*-D-galactopyranoside (IPTG). Followed by IPTG induction, the cells were grown for 4.5 hours. The cells were pelleted down by carrying out centrifugation at 6000 rpm for 20 minutes at 4 °C. This was followed by re-suspension of the pellet in pre-chilled lysis buffer (20 mM Tris–HCl + 500 mM NaCl, pH 8.0). After thorough re-suspension in lysis buffer, the cells were sonicated (25 pulses, each of 30 s pulse time, and an interim time frame of 1 min). Unbroken cells and debris were removed by another round of centrifugation at 10,000 rpm for 10 min. The soluble fraction obtained thereafter was carefully removed and allowed to bind to Ni–NTA agarose resin. The Ni–NTA column was washed using 50 ml wash buffer (20 mM Tris–HCl, 500 mM NaCl and 50 mM imidazole, pH 8.0) followed by elution with 20 mM Tris–HCl, 500 mM NaCl and 500 mM imidazole, pH 8.0. The eluted fractions were pooled according to their tentative protein content as per their absorbance at 280 nm. The post-elution fractions were subjected to dialysis in 20 mM Na-phosphate buffer pH 7.5. The activity assay performed with the purified protein showed that HPPK is catalytically active. We further overexpressed and purified three catalytically inactive mutants of HPPK viz. P43A, L45A and N55A. Catalytic sites were selected based on previously published works on pterin binding^15,25,26^. The pET28A plasmids overexpressing the inactive HPPK mutants were obtained from Genescript and overexpression was carried out using the above-mentioned protocol.

### HPPK activity assay

The HPPK activity assay was conducted with the help of KinaseGlo^TM^ assay kit. In this assay, firefly luciferase utilizes the ATP remaining after HPPK catalysis to produce a luminescence signal that is directly proportional to ATP concentration; from this, the HPPK activity can be derived. The enzyme activity calculation and selection of optimum concentration was done following previously published methods^15^. For kinetic measurements, an optimized HPPK concentration of 7 ng/50 µL assay volume was determined, which allowed for monitoring the first 10% of reactions turnover in a reasonable assay time period (20 min).

Measurements were performed in 96-well plates using assay buffer (100 mMTris-HCl/10 mM MgCl_2_, pH 8.5, 0.01% (w/v) BSA, 0.01% (v/v) Tween 20 and 10 mM β-mercaptoethanol). Typically, 5 µl of test compound (dissolved in 50% DMSO) and 20 µl of enzyme were added to each well followed by 25 µl of assay buffer giving 18 µM pterin and 2 µM ATP in a total reaction volume 50 µl. After a 20-minute incubation at room temperature, the enzymatic reaction was stopped with 50 µl of KinaseGlo™ reagent. Luminescence was recorded after a further 10 min using the plate reader (Tecan Infinite M200 Pro). The inhibition constant K_i_ was derived using the relation ${Ki}=\frac{{IC}50}{1+\left[S\right]/{Km}}\,$. All the measurements were conducted in triplicate, and the error values are indicated by standard errors.

### Differential interference contrast (DIC)

WT Escherichia coli BW25113 cells were grown in M9 media supplemented with 0.8gL^−1^ glucose and casamino acids (mixtures of all amino acids except tryptophan) in absence and presence of CD15-3 at 42 °C for incubation and 300 rpm constant shaking. A drop in DHFR activity has been associated with cellular filamentation and a similar phenotype is observed under TMP treatment^23^. Since CD15-3 targets intracellular DHFR and soluble fraction of cellular DHFR is lower at 42 degrees C we chose this temperature for our imaging studies^45^.

Aliquots were taken from the growing culture, and they were drop casted on agar bed/blocks. These blocks were taken further processed for differential inference contrast (DIC) imaging using Zeis Discovery imaging workstation. Multiple fields were observed and scanned for a single condition type and a minimum of three replicates were used for imaging studies. Similar methods for imaging were used for WT Escherichia coli BW25113 cell types overexpressing *folK* under conditions of absence and presence of CD15-3 compound. Intellesis Module was used for analyzing DIC images. On average, around 500 cells were analyzed for computing cell length. *E. coli* cell lengths in our imaging studies were not normally distributed. Nonparametric Mann-Whitney test was therefore used to determine if the cell length distributions were significantly different upon CD15-3 treatment.

### Reporting summary

Further information on research design is available in the Nature Portfolio Reporting Summary linked to this article.

## Supplementary information

Supplementary Information

Peer Review File

Description of Additional Supplementary Files

Supplementary Data 1

Reporting Summary

## Data Availability

The source data underlying Figs. 2B, C, 4A, 6A, B, D, Supplementary Fig. 3A, D, E, Supplementary Fig. 5A–E, Supplementary Fig. 6A–F and Supplementary Fig. 9A, B are provided as a Source Data file. The data for the metabolomics analyses and metabolic modelling are provided as Supplementary Data 1. An extended version of this file, also including all information relevant for metabolite identification, is available at 10.6084/m9.figshare.22583869. The raw mass spectrometry data of the metabolomics experiments have been uploaded to MetaboLights (https://www.ebi.ac.uk/metabolights/) with accession code MTBLS6092 and to 10.6084/m9.figshare.22583869. The following published protein structures were used in this study: 1ET0, 1AJ0, 1W7K, 1DRA, 1JYS, 1B0A, 4S2U, 1M3U, 1ZP3, 4M5I, 1T3D. Source data are provided with this paper.

## Source data

Source Data
