# Supplementary material for: Empowering drug off-target discovery with metabolic and structural analysis
Source: Nat Commun. 2023 Jun 9;14:3390. doi: 10.1038/s41467-023-38859-x (PMC10256842; doi:10.1038/s41467-023-38859-x)
Supplement: Supplementary file 1 — Supplementary Information [file 41467_2023_38859_MOESM1_ESM.pdf]

**Supplementary Information (SI) Guide**

**Supplementary Note 1:** The case when 2 enzymes are inhibited by a common inhibitor.

**Supplementary Figures**

- **Supplementary Figure 1:** Comparative growth curves for WT [WT Escherichia coli BW25113] control and CD15-3 treated cells.
- **Supplementary Figure 2:** Growth curves for WT [WT Escherichia coli BW25113] control and WT cells, pre-exposed to CD15-3.
- **Supplementary Figure 3:** Metabolites selected for external supplementation experiments did not show any toxic effect on the growth profiles of the cells.
- **Supplementary Figure 4:** Correlation of metabolic supplementation induced growth rescue under CD15-3 treatment with model calculated growth benefit.
- **Supplementary Figure 5:** Effect of overexpression of select folate pathway genes on CD15-3 treated cells.
- **Supplementary Figure 6:** Effect of overexpression of select folate pathway genes on Trimethoprim (TMP) treated cells.
- **Supplementary Figure 7:** Summary of selected supplement and overexpression evidence for a folate-related mechanism of action of CD15-3.
- **Supplementary Figure 8:** DIC image showing overexpression of folK rescues cells from CD15-3 induced morphological changes.
- **Supplementary Figure 9:** Results of chemiluminescence assays showing CD15-3 interacts with HPPK.

**Supplementary Table 1:** Molecular Docking Results

**Supplementary Note 1**

**The case when 2 enzymes are inhibited by a common inhibitor.**

Here we consider the situation when two enzymes are inhibited by common inhibitor. We use the following notations:

$x_0$ total concentration of the inhibitor (i.e. free + bound)
$x_{free}$ concentration of free inhibitor in solution
$E_1^0$ total concentration of enzyme 1 in the cell
$E_1^{free}$ concentration of free (unbound) enzyme 1 in the cell
$[E_1x]$ concentration of bound enzyme 1-inhibitor complex in the cell
$E_2^0$ total concentration of enzyme 2 in the cell
$E_2^{free}$ concentration of free (unbound) enzyme 2 in the cell
$[E_2x]$ concentration of bound enzyme 2-inhibitor comnplex in the cell
$K_{d1}, K_{d2}$ binding affinities of enzyme 1 and 2 respectively to the inhibitor

Using Law of Mass Action and conservation of mass for all three components (enzymes 1 and 2 and the inhibitor) we get for both enzymes:

$$[E_1x] = \frac{E_1^{free}x_{free}}{K_{d1}} \qquad (2)$$

$$[E_2x] = \frac{E_2^{free}x_{free}}{K_{d2}} \qquad (3)$$

$$[E_1x] + [E_2x] + x_{free} = x_0 \qquad (4)$$

$$[E_1x] + E_1^{free} = E_1^0 \qquad (5)$$

$$[E_2x] + E_2^{free} = E_2^0 \qquad (6)$$

$$E_1^0 - E_1^{free} = \frac{E_1^{free}x_{free}}{K_{d1}};\ E_1^{free} = \frac{E_1^0}{1+\frac{x_{free}}{K_{d1}}};\ [E_1x] = \frac{E_1^0x_{free}}{x_{free}+K_{d1}} \qquad (7)$$

$$E_2^0 - E_2^{free} = \frac{E_2^{free}x_{free}}{K_{d2}};\ E_2^{free} = \frac{E_2^0}{1+\frac{x_{free}}{K_{d2}}};\ [E_2x] = \frac{E_2^0x_{free}}{x_{free}+K_{d2}} \qquad (8)$$

$$\frac{E_1^0x_{free}}{x_{free}+K_{d1}} + \frac{E_2^0x_{free}}{x_{free}+K_{d2}} + x_{free} = x^0 \qquad (9)$$

$$x_{free}^3 + x_{free}^2(E_1^0 + E_2^0 + K_{d1} + K_{d2} - x_0) + x_{free}\big(E_1^0K_{d2} + E_2^0K_{d1} + K_{d1}K_{d2} - x_0(K_{d1} + K_{d2})\big) - x_0K_{d1}K_{d2} = 0 \qquad (10)$$

Now consider the overexpression case $E_1^0 \gg E_2^0;\ K_{d1} \approx K_{d2}$ i.e. $\frac{E_1^0x_{free}}{x_{free}+K_{d1}} \gg \frac{E_2^0x_{free}}{x_{free}+K_{d2}}$

This inequality means that most of the inhibitor is bound to enzyme 1 (naturally as it is overexpressed). In this case the cubic equation for free concentration becomes quadratic because we neglect in the first approximation the inhibitor bound to enzyme 2

$$E_1^0 x_{free} + {x_{free}}^2 + K_{d1} x_{free} = x_0 x_{free} + K_{d1} x_0 \qquad (11)$$

$$ {x_{free}}^2 + x_{free}(E_1^0 + K_{d1} - x_0) - K_{d1} x_0 = 0 \qquad (12)$$

$$x_{free} = \frac{-(E_1^0 + K_{d1} - x_0) + \sqrt{(E_1^0 + K_{d1} - x_0)^2 + 4K_{d1}x_0}}{2} \qquad (13)$$

assuming high overexpression

$$E_1^0 \gg x_0, K_{d1} \qquad (13)$$

$$x_{free} \approx \frac{x_0 K_{d1}}{E_1^0} \qquad (14)$$

$$E_2^{free} = \frac{E_2^0}{1 + \frac{x_0 K_{d1}}{E_1^0 K_{d2}}} \qquad (15)$$

Thus, the effect of overexpression of one enzyme affects the way that another enzyme is inhibited by the same inhibitor in the positive way: the more one enzyme is overexpressed the less other enzyme is inhibited because the inhibitor is sequestered into a binding complex with the overexpressed enzyme. However, with respect to increased expression of the inhibitor we can see 2 regimes:

when overexpression is high $\frac{K_{d2} x_0}{K_{d1} E_1^0} \ll 1$.

In this case

$$E_2^{free} = \frac{E_2^0}{1 + \frac{x_0 K_{d1}}{E_1^0 K_{d2}}} \approx E_2^0 \left(1 - \frac{x_0}{E_1^0}\frac{K_{d1}}{K_{d2}}\right) \approx E_2^0 \qquad (16)$$

i.e. fitness does not depend on the inhibitor concentration because the overexpressed enzyme sequesters most of the inhibitor and the other target enzyme can function uninhibited.

In the opposite case of high concentration of the inhibitor relatively to the overexpressed enzyme or when binding affinities differ strongly between two enzymes, residual inhibitor can inhibit second (not overexpressed) enzyme.

This scenario corresponds to the case $\frac{K_{d2} x_0}{K_{d1} E_1^0} \gg 1$ i.e., when there is plenty of inhibitor - more than overexpressed enzyme can bind - the approximation is different, and the result is

$$x_{free} \approx x_0 - E_1^0 \qquad (17)$$

$$E_2^{free} = \frac{E_2^0}{1 + \frac{x_0 - E_1^0}{K_{d2}}} \approx \frac{E_2^0}{\frac{x_0 - E_1^0}{K_{d2}}} \qquad (18)$$

We conclude that there can be an asymmetry between the effect of overexpression of HPPK and DHFR. If HPPK is highly overexpressed such that it sequesters most of the inhibitor, then the fitness – concentration of the inhibitor curve will be flat. DHFR overexpression from pBAD was restricted to 0.005% arabinose induction to avoid overexpression induced toxicity [1] which would complicate interpretation of the rescue experiments. If DHFR is less expressed from the plasmid, i.e. at lower concentration of arabinose as in our experiment (0.005% for folA vs 0.1% for HPPK) then upon addition of inhibitor the inhibitor-growth will be flat at lower concentration of the inhibitor followed by loss of fitness at higher concentration of the inhibitor due to inhibition of HPPK by remaining inhibitor. Inhibition of growth occurs in this case when concentration of the inhibitor becomes greater or equal to concentration of the overexpressed protein. E.g., 1000-fold overexpression of DHFR equals to about 100 micromoles concentration in the cell – roughly similar to the concentration of CD15-3 in the experiments. We propose HPPK overexpression associated rescue does not directly mean that HPPK is more of a 'primary' target compared to DHFR; rather it is just that we could not fully scan DHFR overexpression induced phenotype and overexpression of either enzyme would lead to sequestration the drug thereby alleviating CD15-3 induced growth inhibition.

**Supplementary Figures:**

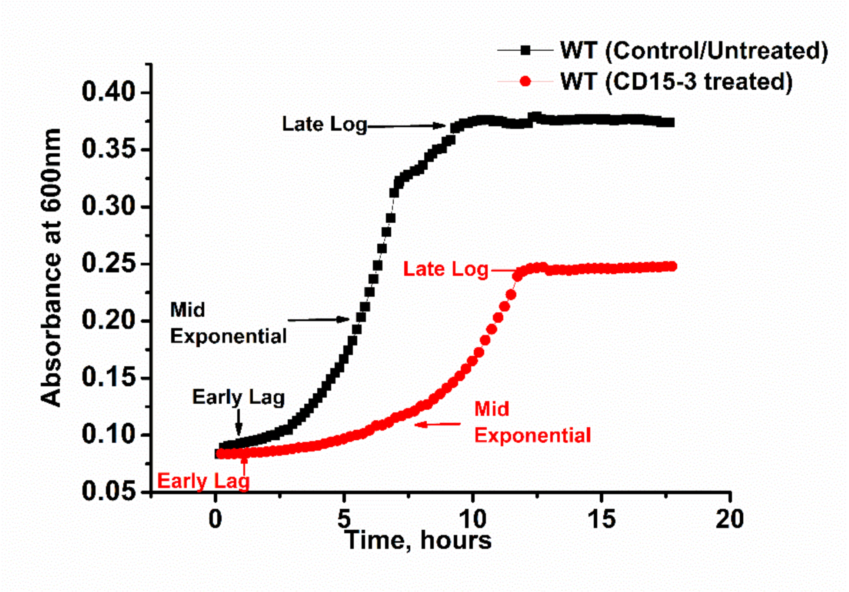


**Supplementary Figure 1:** Comparative growth curves for WT [WT Escherichia coli BW25113] control (black trace) and CD15-3 treated cells (red trace). Respective phases at which cells were harvested are indicated for both WT untreated control and CD15-3 treated cells. Cells were harvested for metabolomics experiments at their respective early lag phase (~30 minutes for WT untreated control and ~45 minutes for WT+CD15-3 treated), mid exponential phase (~ 5 hours for WT untreated control and ~6.5 hours for WT+CD15-3 treated) and late log phase (~10 hours for WT untreated control and ~12 hours for WT+CD15-3 treated).

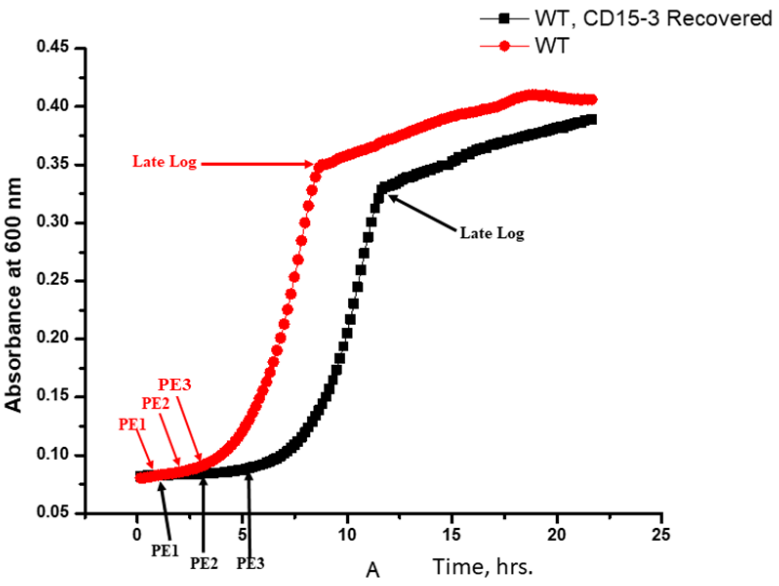


**Supplementary Figure 2:** Growth curves for WT [WT Escherichia coli BW25113] (naïve in red) and WT pre-exposed cells (CD15-3 recovered in black). PE1, PE2 and PE3 are respectively pre-exponential stages 1, 2 and 3 of growth curve. Stages (PE1, PE2, PE3 and Late Log) marked in red are for naïve WT cells and those marked in black refer to pre-exposed WT. For the recovery assay experiments, naïve and CD15-3 pre-exposed cells were harvested at their respective pre-exponential and late log phases of growth.

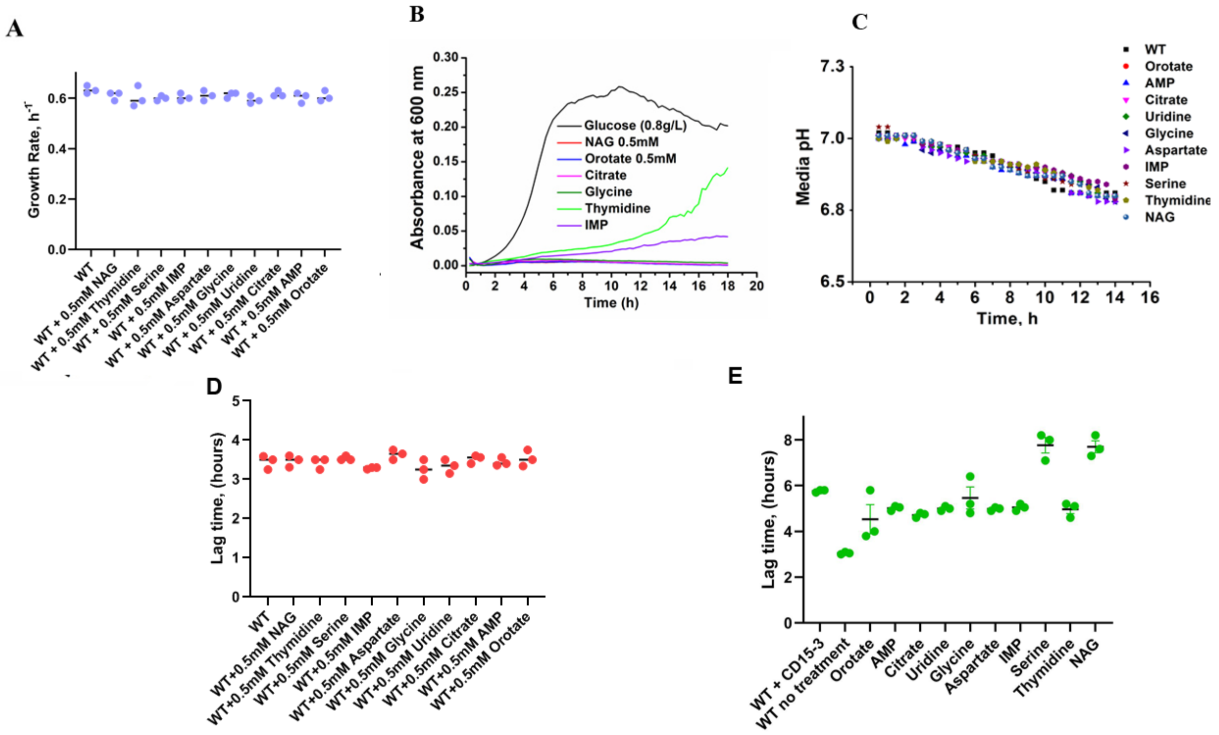


**Supplementary Figure 3:** Metabolites selected for external supplementation experiments did not show any toxic effect on the growth profiles of the cells. (A) Bar plot showing growth rates of WT cells [WT Escherichia coli BW25113] grown in the presence of metabolites as external supplements in M9 medium with glucose at 0.8 g/L. Growth rates were derived from 3 biologically independent bacterial (WT Escherichia coli BW25113) cell cultures. (B) Growth curve shows that these external metabolites did not show any potential to function as an alternate C-source in the growth media. (C) Media (M9 media with glucose at 0.8 g/L) pH were monitored all throughout the culture time, when cells were allowed to grow in metabolite supplemented media (one metabolite at a time). Metabolites at the tested concentration of 0.5milli-Molar do not result in change of media pH. (D) Dot plot shows that metabolite supplementation at the tested concentration do not alter the growth lag-time in the absence of stress (induced with CD15-3 in our study). Lag times were derived from 3 biologically independent bacterial (WT Escherichia coli BW25113) cell cultures. (E) Bar plot showing variation in lag-time during metabolite supplementation experiments. NAG and serine supplementation leads to a prolonged lag time compared to other metabolites. Lag times were derived from 3 biologically independent bacterial (WT Escherichia coli BW25113) cell cultures. Data are presented as mean values +/- SEM. Source data are provided as a Source Data file.

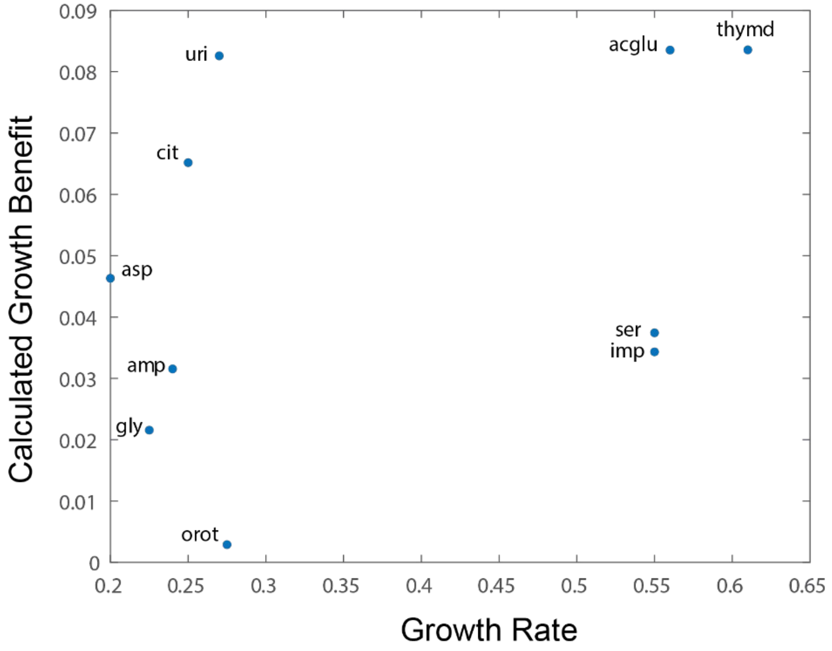


**Supplementary Figure 4:** Correlation of metabolic supplementation induced growth rescue under CD15-3 treatment with model calculated growth benefit. Flux balance analysis was used to calculate a maximum growth flux solution under glucose growth, and solution shadow prices were extracted for each metabolite. Shadow prices represent the benefit of each metabolite to the FBA objective, which is growth. An overall correlation is observed, including for metabolites not listed in the main text, namely aspartate and n-acetyl glutamate, demonstrating a rationale for the ability of these metabolites to rescue growth.

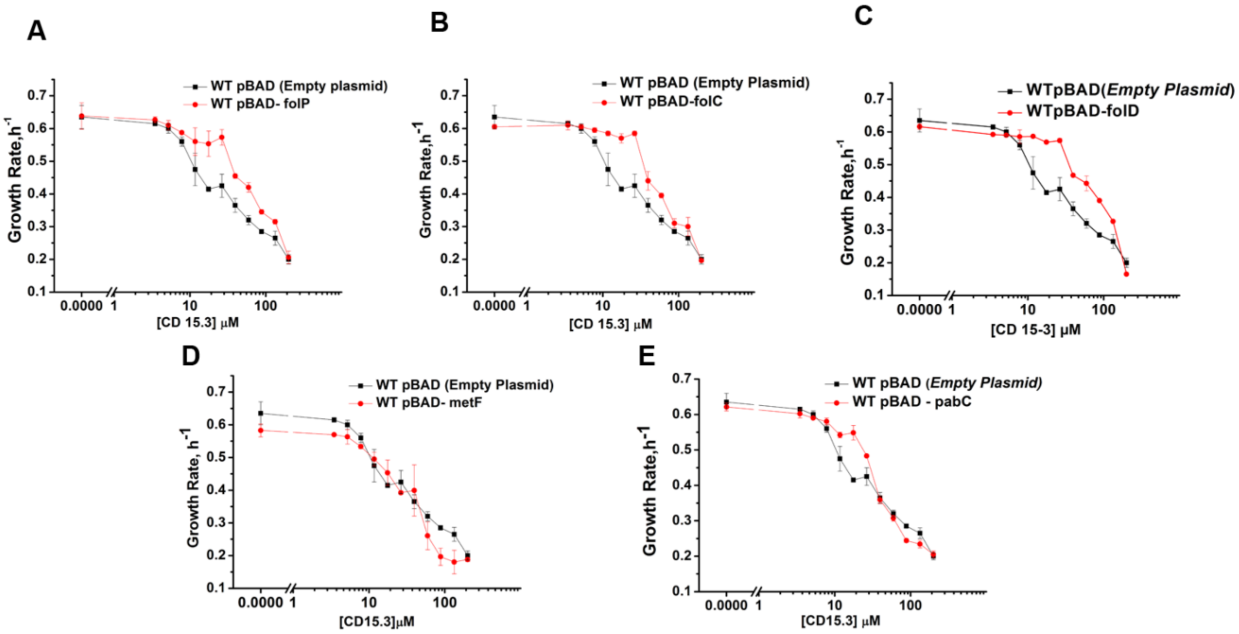


**Supplementary Figure 5:** Overexpression of (A) folP (encoding DHPS) (B) folC (encoding DHFS) and (C) fol D (encoding MTHFC) showed slight improvement in the growth rates of the CD15-3 treated cells only at lower concentrations (<50 µM) of CD15-3. folP, folC and folD genes were overexpressed at 0.1% arabinose induction under pBAD promoter. (D) Overexpression of metF (encoding MTHFR) under pBAD promoter with 0.1% arabinose induction did not lead to recovery from CD15-3 induced growth inhibition. (E) Overexpression of ADCL (encoded by gene pabC) under pBAD promoter with 0.1% arabinose induction was found to have no recovery effect on CD15-3 treated cells. Growth rates for control (pBAD empty plasmids) and each of the conditions tested (over-expressions) were derived from at three biologically independent bacterial (WT Escherichia coli BW25113) cell cultures. All the data are presented as mean values +/- SEM. Source data are provided as a Source Data file.

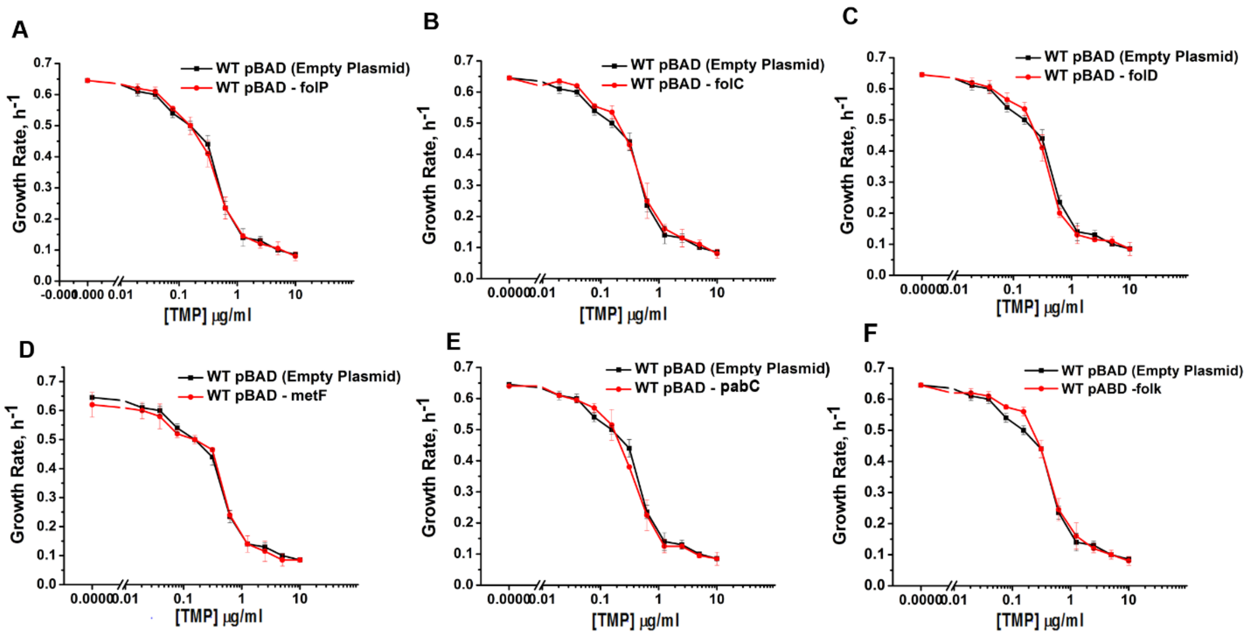


**Supplementary Figure 6:** Overexpression of (A) folP (encoding DHPS) (B) folC (encoding DHFS) (C) fol D (encoding MTHFC) (D) metF (encoding MTHFR) (E) ADCL (encoded by gene pabC) and (F) HPPK (enocoded by folK) was found to have no recovery effect on Trimethoprim (TMP) treated BW27783 cells. The overexpressed genes were under pBAD promoter, and the overexpression was induced with 0.1% arabinose. Growth rates for control (pBAD empty plasmids) and each of the conditions tested (over-expressions) were derived from at three biologically independent bacterial (WT Escherichia coli BW25113) cell cultures. All the data are presented as mean values +/- SEM. Source data are provided as a Source Data file.

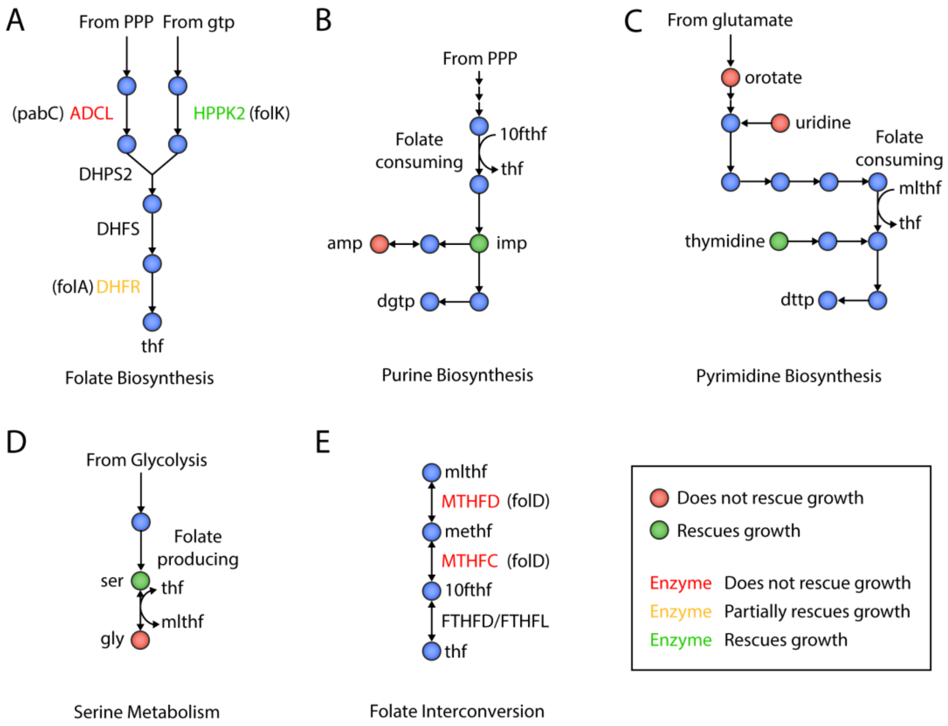


**Supplementary Figure 7:** Summary of selected supplement and overexpression evidence for a folate-related mechanism of action of CD15-3. A) De novo folate biosynthesis. Three enzymes were overexpressed. Overexpression of the intended targeted, folA, partially restored growth. Overexpression of pabC (adcl) did not restore growth. B) Purine biosynthesis. IMP and AMP metabolic supplementation was done. IMP supplementation rescued growth while AMP had no effect in rescuing CD15-3 induced growth inhibition. C) Pyrimidine biosynthesis. Thymidine supplementation led to growth rescue from CD15-3 induced inhibition and Orotate and uridine had no effect in recovery. D) Serine biosynthesis. Metabolic supplementation of serine resulted in growth rescue. E) Folate interconversion.

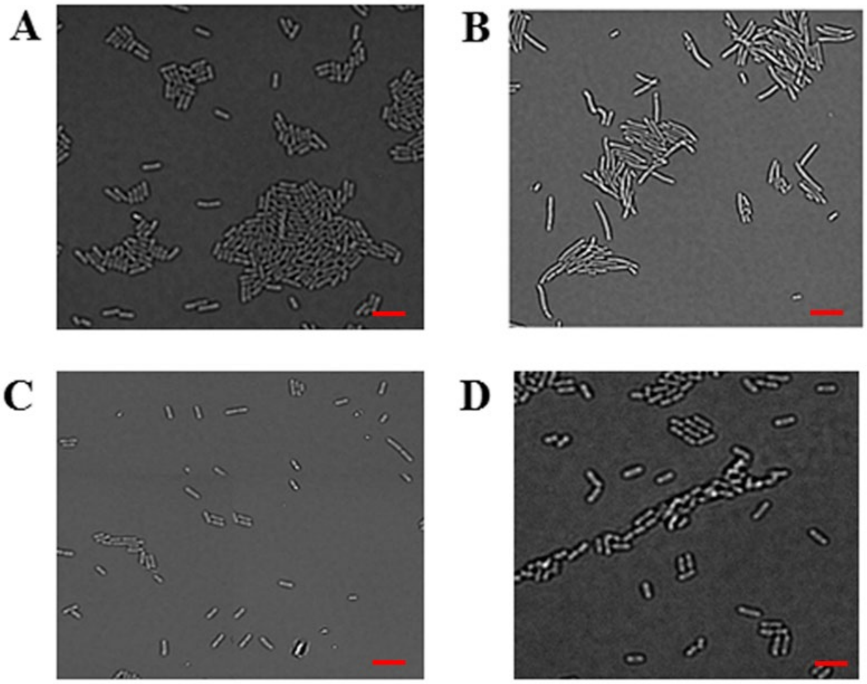


**Supplementary Figure 8:** Overexpression of folK rescues cells from CD15-3 induced morphological changes. DIC image of WT [WT Escherichia coli BW25113] cells under (A) control (no CD15-3 treatment) and (B) treated (CD15-3 treatment) conditions. CD15-3 treated cells shows visible signs of cellular filamentation. (C) DIC image of WT E. coli cells overexpressing folK under control (no CD15-3 treatment) and (D) CD15-3 treated condition. Unlike WT cells (B) showing cellular filamentation in presence of CD15-3, folK overexpressing cells upon CD15-3 treatment did not manifest visible signs of filamentation. Scale bar corresponds to a cell length of 2 μm. Imaging experiments were carried out for at least 4 times for each of the test conditions as well as the control and representative images for each of the control and experimental conditions have been shown here.

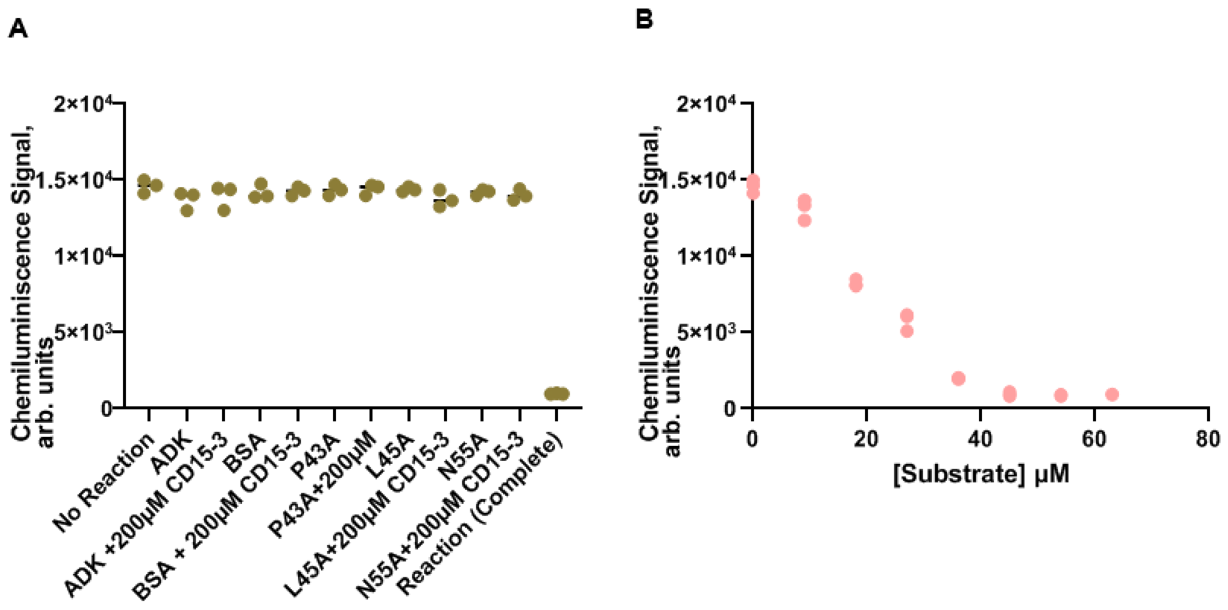


**Supplementary Figure 9: (A)** Bar plot showing the range of chemiluminescence signal at zero substrate (no reaction) condition and the end of reaction (no inhibitor in the assay buffer). Absolute chemiluminescence signal intensity as monitored under different control conditions. ADK was used in the assay as the unrelated kinase (non-substrate of HPPK) and BSA along with three catalytically inactive mutants of HPPK viz P43A, L45A and N55A were used as the negative control proteins in the assay. Catalytically inactive mutants were selected based previous reports of HPPK substrate binding [2-4]. No drop in the chemiluminescence signal was observed in the negative control reaction sets. Similar readout was also found with BSA (a non-ATP using protein) Adk (an ATP dependent non-substrate protein for HPPK) and the three catalytically inactive mutants of HPPK in presence of CD15-3 in the assay buffer. (B) Drop in chemiluminescence signal was observed when substrate concentration was kept increasing, keeping inhibitor (CD15-3) concentration constant at ~40 µM. arb. units. in the figures 9A and B refers to arbitrary unit. n=3 biologically independent experiments were carried out. Source data are provided as a Source Data file.

**Supplementary Table 1: Molecular Docking Results:**

| Target Protein/Encoding Gene | Binding Efficiency (kcal/mol) |
|---|---|
| DHPS/folP | -8 |
| DHFS/folC | -9.4 |
| MTHFD/metF | -5.6 |
| ADCL/pabC | -5.9 |
| HPPK/folK | -12 |
| folD | -9 |

## Supplementary References


1 Bhattacharyya, S. *et al.* Transient protein-protein interactions perturb E. coli metabolome and cause gene dosage toxicity. *elife* **5**, e20309 (2016).

2 Chhabra, S. *et al.* Structure of S. aureus HPPK and the discovery of a new substrate site inhibitor. *PloS one* **7**, e29444 (2012).

3 Marimuthu, P., Singaravelu, K. & Namasivayam, V. Probing the binding mechanism of mercaptoguanine derivatives as inhibitors of HPPK by docking and molecular dynamics simulations. *Journal of Biomolecular Structure and Dynamics* **35**, 3507-3521 (2017).

4 Shi, G., Blaszczyk, J., Ji, X. & Yan, H. Bisubstrate analogue inhibitors of 6-hydroxymethyl-7, 8-dihydropterin pyrophosphokinase: synthesis and biochemical and crystallographic studies. *Journal of medicinal chemistry* **44**, 1364-1371 (2001).
