# Supplementary material for: Empowering drug off-target discovery with metabolic and structural analysis
Source: Nat Commun. 2023 Jun 9;14:3390. doi: 10.1038/s41467-023-38859-x (PMC10256842; doi:10.1038/s41467-023-38859-x)
Supplement: Supplementary file 3 — Description of Additional Supplementary Files [file 41467_2023_38859_MOESM3_ESM.pdf]

Description of Additional Supplementary Files

Supplementary Data 1 : Tables containing metabolomics data for CD15-3 and other antibiotics, metabolite ID mapping, Structural clustering analysis, and metabolite transporter presence.
