# Supplementary material for: Empowering drug off-target discovery with metabolic and structural analysis
Source: Nat Commun. 2023 Jun 9;14:3390. doi: 10.1038/s41467-023-38859-x (PMC10256842; doi:10.1038/s41467-023-38859-x)
Supplement: Supplementary file 2 — Peer Review File [file 41467_2023_38859_MOESM2_ESM.pdf]

nature portfolio

Peer Review File

Empowering systems-guided drug off-target discovery with metabolic and structural analysis

Open Access This file is licensed under a Creative Commons Attribution 4.0 International License, which permits use, sharing, adaptation, distribution and reproduction in any medium or format, as long as you give appropriate credit to the original author(s) and the source, provide a link to the Creative Commons license, and indicate if changes were made. In the cases where the authors are anonymous, such as is the case for the reports of anonymous peer reviewers, author attribution should be to 'Anonymous Referee' followed by a clear attribution to the source work. The images or other third party material in this file are included in the article's Creative Commons license, unless indicated otherwise in a credit line to the material. If material is not included in the article's Creative Commons license and your intended use is not permitted by statutory regulation or exceeds the permitted use, you will need to obtain permission directly from the copyright holder. To view a copy of this license, visit http://creativecommons.org/licenses/by/4.0/.

REVIEWER COMMENTS

Reviewer #1 (Remarks to the Author):

In this study, titled 'Empowering systems-guided drug target discovery with metabolic and structural analysis', Chowdhury and Zielinski et al. build on previous work from their group that identified and characterised CD15-3. This compound was designed against bacterial DHFR (folA) and can inhibit wild type as well as trimethoprim-resistant alleles of the enzyme. Their earlier studies showed that cells overproducing DHFR are only partially resistant to CD15-3. The authors interpret this to mean that CD15-3 has additional targets in E. coli. In order to identify these targets, the authors use metabolomics approaches to first identify specific metabolites that are altered upon CD15-3 treatment, and when supplemented into the growth medium, have the potential to rescue bacterial growth in the presence of the inhibitor. Next, they map these metabolites onto biochemical pathways operative in E. coli to show that folate metabolism is the most crucially inhibited pathway upon CD15-3 treatment. The authors interpret this to mean that an additional target of this inhibitor is likely to be present in the folate metabolism pathway itself. Using structural similarities to DHFR, they identify some possible targets of CD15-3, and use overexpression and growth rescue experiments to discover that the enzyme HPPK (folK) is likely to be an additional target of CD15-3. Lastly, they show that presence of CD15-3 has an inhibitory effect on HPPK activity in vitro in cell-free lysates.

My assessment of the strengths and weaknesses of this study are as follows:

Strengths:

1. This study addresses an important lacuna in the area, namely the discovery of drug-targets in bacteria. It is established that the most effective antibiotics tend to target more than a single enzyme in bacteria. Yet, clear pipelines for the discovery of additional targets haven’t been established yet. This study attempts to fill this gap.

2. Anti-folate compounds are widely used drugs for a variety of diseases. This paper uncovers a novel aspect of an anti-folate compound, as an inhibitor of HPPK.

3. This study uses a powerful combination of computation and experiment to attempt to discover new biology.

4. Given the multi-disciplinary nature of the study, I acknowledge how well-written the paper is as well as the presentation of data, which make the study accessible to a wide readership.

Weaknesses:

1. This study focuses on CD15-3, which is a compound that the authors have reported recently, but is neither used clinically, nor a standard drug that is accessible or used by others. Thus, the applicability of this study to the wider understanding of anti-folates is limited.

2. Despite the promise of the approaches used here, the final set of experiments to establish HPPK as the target of CD15-3 could have been shored up with some more experiments (outlined later in my note).

3. The finding that a compound designed to bind to the active site of DHFR inhibits another enzyme that accommodates chemically similar compounds is not in itself exciting. Given the multi-disciplinary nature of the techniques used in this study, it might have been more effective to apply them to other antibiotics, for which metabolic signatures are less restricted than anti-folates. The authors' own admission to this is noted, but does not sufficiently diminish the power of their pipeline.

Based on the above, I believe that the value of this study in its present form lies in the methods used and the questions being asked, rather than the results of the study themselves. This should not however preclude the study from being published in Nature Communication. However, the following questions and comments should be addressed before the paper is accepted for publication.

1. HPPK inhibition assay: Since the authors have used an indirect enzyme-linked assay to assess the activity of HPPK, it is imperative that they validate the assay with a catalytically inactive mutant of HPPK. This is particularly relevant since the authors have only used diluted lysates, and not pure HPPK for their assay. BSA control is in my opinion insufficient here.

2. A complementary approach to the above would be to show that the inhibition of CD15-3 on HPPK in the assay is competitive (presumably) and can be surmounted by a large excess of specific substrate.

3. Other folate metabolism genes such as folP were also found to partially diminish the effects of CD15-3. Is it likely that CD15-3 is a generic inhibitor for multiple enzymes in the pathway? It would be worth testing (and perhaps negating) a few of these other hits computationally/biochemically.

4. The authors use the data for folK and folA overexpression (and other fol enzymes also) to make the claim that folK is the primary target of CD15-3. This would only hold true if both enzymes were overexpressed to similar extents. This represents an important missing control and should be addressed.

5. Earlier results from this group demonstrate that folA overexpression leads to growth inhibition in E. coli. Could this not contribute to the inadequate growth rate recovery in CD15-3 treated folA-overexpressing bacteria?

6. Have the authors attempted to dock CD15-3 into the active site of HPPK and other fol enzymes? These results would supplement their biochemical findings well.

7. Ki values of CD15-3 for HPPK and DHFR should be compared to understand why HPPK may be the more important target, rather than the originally intended DHFR.

8. I am unable to reconcile the very similar values of IC50 for bacterial inhibition by CD15-3 and IC50 for HPPK enzyme inhibition by CD15-3. This is surprising because it suggests that a) CD15-3 concentrations outside and inside the cell equilibrate rapidly to become equal. This is almost impossible in Gram negative bacteria. b) if CD15-3 does indeed have multiple targets in E. coli, then the drug IC50 for bacterial growth is very unlikely to the equal to the IC50 for HPPK inhibition.

9. In order to make this study applicable to anti-folates in general, I would recommend using the overexpression assay to test how folk, folP etc influence growth in trimethoprim. A comparison of why CD15-3 behaves similarly/differently from trimethoprim would be a useful addition to this study.

Reviewer #2 (Remarks to the Author):

Chowdhury et al. present a workflow to determine the target of antibiotics. This topic is of great interest considering the urgent need for improving antibiotics discovery and that even some well-known antibiotics have unknown or poorly defined mode of actions. The integrative and systemic workflow proposed gathers metabolomics, growth assays with nutrient supplementations, functional analyses as well as machine learning, constraint-based modeling and structural analyses. The authors provide a “proof of concept” of their workflow on a drug recently designed by the team.

The amount of work is impressive and is a good example of a multi-disciplinary approach in the study of a drug target. However, several issues were found in the results section of the manuscript, and the reviewer encourages a strong rework on different parts of the study to make it suitable for publication. The first comes from the fact that the whole study was made on a drug for which an initial target was already known (dihydrofolate reductase), and this seems to have motivated the authors to focus, on each part of the workflow, on folate metabolism instead of keeping a real "systemic" approach: i) the metabolomics data are only presented for a subpart of the metabolome close to folate metabolism, ii) the machine learning model was used to "discover" that the drug was acting on folate metabolism, which could have been guessed without it, iii) the metabolic model of E. coli is specifically constrained with expression and spontaneous reactions data only around folate metabolism, iv) structural analysis is done on proteins around folate metabolism (structurally close to dihydrofolate reductase). The study is thus a convincing approach to decipher the target(s) of a drug designed to act on folate metabolism, but the authors should do a similar approach on a drug with no a priori on its target to really prove that they developed a systems-guided target discovery workflow. Moreover, some parts of the study lack consistency and rigorous justifications and should be carefully rewritten or reconceptualized. For example, the choice of nutrients of interests seems to have been very arbitrary between the machine learning step and the nutrient supplementation step.

Major comments

Section "Metabolomic analysis of CD15-3 perturbation"

• Displaying (Fig 2A) and commenting only part of the whole metabolomics results is very questionable: only around 50 of the 886 metabolites, and three pathways are shown and commented. How these metabolites were chosen? Are there other abundance changes in other metabolites and pathways? It looks the authors arbitrary chose to only display the pathways that suits the mechanism they will further want to demonstrate, which would make this part of the study biased.

• The differences in metabolite abundances observed at 5h and 12h (Figure 2A) could be due to the differences in growth rates as the presence of the drug slows down growth and metabolic processes, rather than being a direct consequence of the drug mode of action.

• As the carbon source is only provided at 0.8 g/L, it is expected that the non-treated cells will rapidly enter into stationary phase (at 12h, and probably even at 5h depending on the density inoculated). Is it thus pertinent to compare the metabolome of cells that reached stationary phase with metabolome of cells that are still growing? Considering this, only the metabolome at 30 min appears to be relevant. This could notably explain the differences in nucleotide biosynthesis. It should be more relevant to perform metabolomics after 1 or 2 hours of treatment, or to grow the cells with a highest amount of C-source to have a longer exponential growth phase.

• In the 3rd paragraph of the section, the assumption that metabolites displaying the most delayed recovery are the most impacted upon the treatment could be incorrect. Indeed, it also strongly depends on the flux of matter generating the metabolite. It is expected that a central metabolite associated with high matter fluxes, such as pyruvate, will have a fastest recovery than an intermediate metabolite involved in vitamin biosynthesis for example.

• The need to perform the recovery assay (Fig. 2C and 2D and 3rd paragraph of the section) is unclear. Is it an essential part of the main workflow proposed? It doesn't seem so, as it is not directly used in the next part. The authors should emphasize its use in the workflow, or alternatively remove the section.

Section "Machine learning reveals antibiotic mechanism-specific perturbations"

• In the section, the authors show with a LR model that their metabolome profile fits well with the metabolome profile of an antifolate drug. This was strongly expected as the drug was originally designed to interact with dihydrofolate reductase (Zhang et al., 2021). Thus, the quality and relevance of the LR model should be better proven to validate its use in the workflow. For example, the authors could perform similar metabolomics assay on different drugs (with different targets), and test whether the model predicts adequately their class of mechanism.

• How would the proposed workflow work if the drugs have a mode of action different from the five classical mechanisms proposed? As the research for novel antibiotics aims at finding alternatives to classical mode of actions, using an approach only based on data on the classical mode of actions seems irrelevant.

Section "Metabolic modeling predicts patterns in growth rescue experiments for candidate pathway inhibitions"

• The choice of the nutrients used for supplementations and for the subsequent parts of the study is unclear and seems to be arbitrary. IMP was selected from the RL model (Fig 2E), but: i) uacgam, argsuc, cbasp, cys, mnl1p and g3pi were also identified by the model. Using a pragmatic workflow, these should also be included in the supplementation. ii) no clear justification is brought for the inclusion of glycine, serine, thymidine, orotate, uridine is the supplementation assay. As already commented, these arbitrary choices seem to show bias in the study due to a previous idea of the mode of action, rather than a rigourous workflow.

One justification given is that the authors selected metabolites close to folate pathway. This seems a contestable as a drug could target similarly enzymes from very distinct pathways. Furthermore, metabolism is organized as a highly interconnected network rather than by independent pathways. Thus, selecting which metabolites are close or not to a given pathway is difficult to assess pragmatically without a graph-based computational approach.

• 2nd paragraph of the section: Even though the metabolite tested cannot be a sole carbon source, their complementation on a medium with glucose as C-source could boost the growth and induce a higher growth rate. A growth assay of nutrient supplementation on glucose medium should be performed to determine this potential growth rate increase and taken into account before drawing conclusion from Figure 4A. Indeed, the fact that some nutrients increase the growth of treated bacteria could be theoretically independent of the drug mode of action.

• The fact that NAG and serine prolonged the lag phase could be related to an effect on the pH of the medium. The authors should check if adding them change importantly the pH.

• The authors claim that they both tested in silico the effect of nutrient supplementation on i) reaction inhibition ii) cofactor depletion. However, the authors only show one of these results on Figure 3F, 3G, 3H: only one in silico growth is displayed by nutrient. It is unclear which one is depicted. Thus, the claim that “the experimental growth rescue pattern was most consistent with a folate cofactor drain mechanism”. The authors should rework the way they present their metabolic modeling results on this section.

• The authors explain that first, the metabolic models were not able to correctly predict growth rescue with nutrient complementation. They performed several manual modifications of the metabolic model, justified by expression data or spontaneous reactions, to have a better consistency with experimental results. The need to manually modify the models to have a fit with experimental data is questionable and makes the workflow less straightforward than what was expected. This manual modification of the metabolic model seems to have only been done around folate metabolism. It would be preferable to have an automatic way to integrate expression data and spontaneous reactions as mathematical constraints, and then perform the computational analysis on the resulting metabolic model, rather than manually (and potentially with bias) modify what does not fit the authors expectations.

Section “Structural analysis of possible alternate binding targets”

• It is unclear how the authors would have performed the analysis without their a priori knowledge that the drug was designed to target DHFR, as the selection is based on the assumption that the drug should target a protein structurally close to DHFR.

Material and Methods

There is a lack of consistency in the Material and Methods section. The authors should start by giving the species and strain name of the bacteria used (Escherichia coli BW25113) instead of using expressions such as “Bacterial cultures”, “WT cells” “WT BW25113”etc which are confusing.

Also, please clearly refer at the beginning that: M9 medium with glucose at 0.8 g/L was used during all the experiments (unless otherwise stated), as in some part only “M9 medium” is written, which is misleading. Also, the authors should mention clearly which drug concentration(s) where used, and clearly specify if it was not the same in all the results presented, and why.

Same consistency should also be used in figure descriptions and result section.

Minor comments

• Figure 2B and 2C. The Y axis is the same: “Metabolite abundance fold change CD15-3 pre-exposed vs naïve (log scale)” and makes it difficult to understand the difference between the two figures.

• Please improve the consistency in the spelling of “modeling”, that is either spelled modelling or modeling depending the section.

Reviewer #3 (Remarks to the Author):

This manuscript guides you through a workflow that uses metabolomics data in combination with machine learning techniques, metabolic models, and structural analyses to suggest priority candidates for drug target studies.

The workflow was presented rather well at the beginning of the manuscript (Figure 1 and description). The Jupyter notebook that came attached to the manuscript was also highly appreciated. There are however some points that are not entirely clear to me.

1) Firstly, I would like to point out a few things that require immediate rectification prior to publication. The figures in the manuscript (e.g supplementary Figure 4) are of subpar quality and are at many points hard to read. The equations do not carry any equation numbers, making them rather difficult to refer to. Additionally, some equations seem to swap around indices (i.e. subscripts or superscripts for x0). Some equations also have symbols that are not properly rendered, at least in the manuscript that I received. I attached some screenshots that might help.

2) Regarding the machine learning approach presented in the paragraphs: "Machine learning reveals antibiotic mechanism-specific perturbations" and "material and methods". If I understood correctly, a "previously published dataset on the metabolomic response of different antibiotics" was used as a starting point for machine learning to identify specific perturbations of the drug. How is this initial data pool selected (especially for a potential application of this workflow in the future)?

3) There are many regression models available to perform data classification. Why was a multiclass logistic regression model chosen in this study? Does it have any particular advantages?

4) It is also not completely clear to me how the performance of the machine learning model was evaluated. In the manuscript, it was written that default parameters have been used. Following this, the authors carried out a leave-one-out cross-validation. How exactly would the performance be evaluated, if the parameters are kept the same?

5) I would also like to ask a quick question about Figure 2 B. What exactly has been plotted? Fold-change differences between pre-exposed and naive? I have also been told many times that timepoints in metabolomics data can be a quite delicate subject. How have the time intervals been selected?

RESPONSE TO REVIEWER'S COMMENTS

Manuscript Title: Empowering systems-guided drug target discovery with metabolic and structural analysis

Manuscript ID: NCOMMS-22-28234

We sincerely thank all reviewers and the editor for their keen interest in our work and for the valuable comments and suggestions to improve the manuscript. We have revised the article based on the reviewer comments and suggestions. The reviewers mentioned important points and we appreciate the opportunity to clarify these points and include additional experiments for the improvement of our manuscript. Necessary changes as per the comments have been made in the manuscript at appropriate places.

REVIEWER COMMENTS

Reviewer #1 (Remarks to the Author):

In this study, titled 'Empowering systems-guided drug target discovery with metabolic and structural analysis', Chowdhury and Zielinski et al. build on previous work from their group that identified and characterised CD15-3. This compound was designed against bacterial DHFR (folA) and can inhibit wild type as well as trimethoprim-resistant alleles of the enzyme. Their earlier studies showed that cells overproducing DHFR are only partially resistant to CD15-3. The authors interpret this to mean that CD15-3 has additional targets in E. coli. In order to identify these targets, the authors use metabolomics approaches to first identify specific metabolites that are altered upon CD15-3 treatment, and when supplemented into the growth medium, have the potential to rescue bacterial growth in the presence of the inhibitor. Next, they map these metabolites onto biochemical pathways operative in E. coli to show that folate metabolism is the most crucially inhibited pathway upon CD15-3 treatment. The authors interpret this to mean that an additional target of this inhibitor is likely to be present in the folate metabolism pathway itself. Using structural similarities to DHFR, they identify some possible targets of CD15-3, and use overexpression and growth rescue experiments to discover that the enzyme HPPK (folK) is likely to be an additional target of CD15-3. Lastly, they show that presence of CD15-3 has an inhibitory effect on HPPK activity in vitro in cell-free lysates. My assessment of the strengths and weaknesses of this study are as follows:

Strengths:
1. This study addresses an important lacuna in the area, namely the discovery of drug-targets in bacteria. It is established that the most effective antibiotics tend to target more than a single enzyme in bacteria. Yet, clear pipelines for the discovery of additional targets haven't been established yet. This study attempts to fill this gap.
2. Anti-folate compounds are widely used drugs for a variety of diseases. This paper uncovers a novel aspect of an anti-folate compound, as an inhibitor of HPPK.
3. This study uses a powerful combination of computation and experiment to attempt to discover new biology.
4. Given the multi-disciplinary nature of the study, I acknowledge how well-written the paper is as well as the presentation of data, which make the study accessible to a wide readership.

Weaknesses:
1. This study focuses on CD15-3, which is a compound that the authors have reported recently, but is neither used clinically, nor a standard drug that is accessible or used by others. Thus, the applicability of this study to the wider understanding of anti-folates is limited.

Response:

We thank the reviewer for their positive comments and agree that the study does not focus on obtaining a greater understanding of the antifolate drug class in general. However, we believe that as a novel antifolate, CD15-3 reveals the diversity in possible responses to folate perturbation that can be contrasted with the responses to other antifolates such as trimethoprim and sulfamethoxazole. We find it interesting and important that despite inhibiting the same metabolic pathway, the metabolomic and overexpression rescue responses can still be diverse between antifolates.

2. Despite the promise of the approaches used here, the final set of experiments to establish HPPK as the target of CD15-3 could have been shored up with some more experiments (outlined later in my note).

Response:

We thank the reviewer for the suggestion. Based on your recommendation we have performed the HPPK activity assay with purified protein (Figure 6D) and ran control experiments with catalytically inactive variants of HPPK (Supplementary Figure 8A). Three catalytically inactive mutants of HPPK (P43A, L45A and N55A) were used to validate that the drop in signal we observe in presence of CD15-3 in the HPPK reaction-assay set is due to the specific inhibitory interaction between CD15-3 and HPPK. Catalytic sites for mutations were selected based on previously published works on pterin binding (Chhabra et al., 2012; Marimuthu et al., 2017; Shi et al., 2001) . We did not observe any drop in luminescence with BSA, ADK and the three catalytically inactive HPPK mutants' reaction set in the presence of CD15-3. This new result is presented and discussed in *lines 506-515*.

3. The finding that a compound designed to bind to the active site of DHFR inhibits another enzyme that accommodates chemically similar compounds is not in itself exciting. Given the multi-disciplinary nature of the techniques used in this study, it might have been more effective to apply them to other antibiotics, for which metabolic signatures are less restricted than anti-folates. The authors' own admission to this is noted but does not sufficiently diminish the power of their pipeline.

Response:

We agree with the reviewer that applying this pipeline to a broader set of antibiotics would be desirable. We tested the pipeline on the panel of antibiotics from the Zampieri *et.al*. study, but we found that the antifolates had the most consistent metabolic signature, which is logical given that they are the only metabolic inhibitor among that panel of drugs. As a result, we believe that the pipeline is best suited for antimetabolite antibiotics, i.e., those that inhibit growth by inhibiting metabolic enzymes. While many antimetabolite-type antibiotics are discussed in the literature (Zhou et al., 2019), they are not as widely studied as other options and metabolomics data was similarly not available for analysis. There is one study of antibiotic metabolomics that we are aware of that is larger scale than the one we analyzed in this work (Zampieri et al., 2018), but the compound set consists of almost entirely non-metabolic inhibitors.

We hope that the existence of workflows like the one developed in this study motivates large-scale metabolomics analysis of metabolic inhibitors.

Based on the above, I believe that the value of this study in its present form lies in the methods used and the questions being asked, rather than the results of the study themselves. This should not however preclude the study from being published in Nature Communication. However, the following questions and comments should be addressed before the paper is accepted for publication.

1. HPPK inhibition assay: Since the authors have used an indirect enzyme-linked assay to assess the activity of HPPK, it is imperative that they validate the assay with a catalytically inactive mutant of HPPK. This is particularly relevant since the authors have only used diluted lysates, and not pure HPPK for their assay. BSA control is in my opinion insufficient here.

Response:

We thank the reviewer for this suggestion. As mentioned earlier, in the current version we included the HPPK activity assay with purified protein (Figure 6D) and ran control experiments with three catalytically inactive variants of HPPK (Supplementary Figure 8A).

2. A complementary approach to the above would be to show that the inhibition of CD15-3 on HPPK in the assay is competitive (presumably) and can be surmounted by a large excess of specific substrate.

Response:

We thank the reviewer for this interesting suggestion. In the revised manuscript we included the competitive assay using a gradient of substrate. Indeed, with higher concentrations of substrate the CD15-3 induced HPPK inhibition was found to be alleviated with a drop in chemiluminescence signal. (Supplementary Figure 8B).

3. Other folate metabolism genes such as folP were also found to partially diminish the effects of CD15-3. Is it likely that CD15-3 is a generic inhibitor for multiple enzymes in the pathway? It would be worth testing (and perhaps negating) a few of these other hits computationally/biochemically.

Response:

We thank the reviewer for this suggestion. In the current revised version of the manuscript, we included the molecular docking analysis for the select folate pathway proteins with CD15-3. These folate pathway proteins were selected based on structural similarity analysis. Performing biochemical assays with each of these select folate pathway proteins would involve extensive experimentation, and therefore we consider it beyond the scope of the current work. We observed similar trends in binding efficiencies as were observed in the overexpression experiments (the partial rescue observed with some folate pathway genes against CD15-3).

4. The authors use the data for folK and folA overexpression (and other fol enzymes also) to make the claim that folK is the primary target of CD15-3. This would only hold true if both enzymes were overexpressed to similar extents. This represents an important missing control and should be addressed.

Response:

The reviewer is correct that differing levels of overexpression complicates the analysis of which enzyme is the primary target of CD15-3. Indeed, both enzymes are essential to growth and binding of either target inhibits growth.

However, practically speaking we found that folA and folk could not be induced to the same extent. Overexpression of folA upon a certain dosage (beyond the range we used) is toxic to the cells (Bhattacharyya et al., 2016). Thus, folA could still be an important target of the drug, as we have previously demonstrated its binding and inhibition by CD15-3 (Zhang et al., 2021).

We note that in the Supplementary text to the original work we presented a theoretical analysis of the inhibition behavior of the multiple targets of CD15-3 at different concentration regimes of the drug and targets. In this analysis, we demonstrated that overexpression of either target can sequester the drug, alleviating inhibition of both targets.

To summarize, it would be misleading to say that either target is the ‘primary’ target based on the information available. We have gone through the text and could not find language claiming that one target is more important than the other for the function of CD15-3, but we hope the reviewer can point us to any particularly misleading sections.

5. Earlier results from this group demonstrate that folA overexpression leads to growth inhibition in E. coli. Could this not contribute to the inadequate growth rate recovery in CD15-3 treated folA-overexpressing bacteria?

Response:

The reviewer is correct – as we mentioned in the above comment, toxicity upon folA overexpression prevents us from analyzing whether overexpression of folA can completely rescue growth. Due to sequestration of the drug, we would expect that overexpression of any drug-binding enzyme could eventually recover growth, barring toxicity of the overexpression itself. We have attempted to make this point more clear in the current version of the manuscript, in *lines 609-611* of the Discussion.

6. Have the authors attempted to dock CD15-3 into the active site of HPPK and other fol enzymes? These results would supplement their biochemical findings well.

Response:

As mentioned earlier, in the current revised version of the manuscript we included a docking analysis (*Lines 449-451*) and the trends overlap with biochemical findings.

7. Ki values of CD15-3 for HPPK and DHFR should be compared to understand why HPPK may be the more important target, rather than the originally intended DHFR.

Response:

We now performed calculation of Ki of CD15-3 for HPPK and DHFR. We found that CD15-3 has a Ki of 3.54µM against HPPK compared to 5.52 µM against DHFR as was reported in our earlier work. (Zhang et al., 2021) (*Lines 530-533*). The similar Kis could further support that both targets could play a physiological role in growth inhibition.

8. I am unable to reconcile the very similar values of IC50 for bacterial inhibition by CD15-3 and IC50 for HPPK enzyme inhibition by CD15-3. This is surprising because it suggests that a) CD15-3 concentrations outside and inside the cell equilibrate rapidly to become equal. This is almost impossible in Gram negative bacteria. b) if CD15-3 does indeed have multiple targets in E. coli, then the drug IC50 for bacterial growth is very unlikely to the equal to the IC50 for HPPK inhibition.

Response:

We thank the reviewer for this insightful comment. For reference, the IC50 of CD15-3 for culture cells is 72 µM and *in vitro* for HPPK is 39.2 µM. Whether or not this can be considered very similar may be up for debate, but we put forth a few arguments for why this is not particularly strange to us.

The first is that the relative values are quite similar for trimethoprim, which are 1.37µM for culture cells and 0.93µM for an *in vitro* DHFR assay.

The second point is that the relative values for CD15-3 are in the expected ranking, where the IC50 is greater for culture cells where transport has not perfectly equilibrated and binding to non-HPPK targets may be occurring, compared to the *in vitro* assay with overexpressed HPPK.

The third is that there are transporters in *E. coli*, not for complete folates, but for some folate precursors such as p-aminobenzoic acid. So, while we have not annotated the transporter of CD15-3, it is plausible that it could be taken up by promiscuous transporters and thus the IC50 might not be far from what would be expected from equilibrium transport.

We note that the IC50s additionally fall within the expected relative range compared to the Ki values, given by the standard relationship $Ki = IC50/(1+S/Km)$. Given the fact that we used saturating substrate concentrations for the assays, resulting in a denominator >> 1, it is expected that Ki would be substantially below the IC50 as is observed.

Based on these arguments, the calculated parameters seem to be reasonable to us. We would be interested to know if the reviewer had concerns with these arguments that could help us further refine our interpretation of our results.

9. In order to make this study applicable to anti-folates in general, I would recommend using the overexpression assay to test how folk, folP etc influence growth in trimethoprim. A comparison of why CD15-3 behaves similarly/differently from trimethoprim would be a useful addition to this study.

Response:

We thank the reviewer for this interesting suggestion. We performed the suggested experiments involving overexpression of the select genes and probe for recovery signals in cells subjected to Trimethoprim (TMP) induced stress. We did not find any visible sign of growth recovery (other than over a narrow low concentration regime of TMP for cells overexpressing folK) upon overexpressing these genes. We discussed these new results (*lines 451-457*) and included the profiles as a supplementary figure (Supplementary Figure: 5A-F)

Reviewer #2 (Remarks to the Author):

Chowdhury et al. present a workflow to determine the target of antibiotics. This topic is of great interest considering the urgent need for improving antibiotics discovery and that even some well-known antibiotics have unknown or poorly defined mode of actions. The integrative and systemic workflow proposed gathers

metabolomics, growth assays with nutrient supplementations, functional analyses as well as machine learning, constraint-based modeling and structural analyses. The authors provide a “proof of concept” of their workflow on a drug recently designed by the team. The amount of work is impressive and is a good example of a multi-disciplinary approach in the study of a drug target. However, several issues were found in the results section of the manuscript, and the reviewer encourages a strong rework on different parts of the study to make it suitable for publication. The first comes from the fact that the whole study was made on a drug for which an initial target was already known (dihydrofolate reductase), and this seems to have motivated the authors to focus, on each part of the workflow, on folate metabolism instead of keeping a real “systemic” approach: i) the metabolomics data are only presented for a subpart of the metabolome close to folate metabolism, ii) the machine learning model was used to “discover” that the drug was acting on folate metabolism, which could have been guessed without it, iii) the metabolic model of E. coli is specifically constrained with expression and spontaneous reactions data only around folate metabolism, iv) structural analysis is done on proteins around folate metabolism (structurally close to dihydrofolate reductase). The study is thus a convincing approach to decipher the target(s) of a drug designed to act on folate metabolism, but the authors should do a similar approach on a drug with no a priori on its target to really prove that they developed a systems-guided target discovery workflow. Moreover, some parts of the study lack consistency and rigorous justifications and should be carefully rewritten or reconceptualized. For example, the choice of nutrients of interests seems to have been very arbitrary between the machine learning step and the nutrient supplementation step.

Major comments

Section “Metabolomic analysis of CD15-3 perturbation”

• Displaying (Fig 2A) and commenting only part of the whole metabolomics results is very questionable: only around 50 of the 886 metabolites, and three pathways are shown and commented. How were these metabolites chosen? Are there other abundance changes in other metabolites and pathways? It looks the authors arbitrary chose to only display the pathways that suits the mechanism they will further want to demonstrate, which would make this part of the study biased.

Response:

We thank the reviewer for raising this point. In our heat map capturing metabolomic perturbations we showed 50 plus metabolites which in general represent major metabolic processes associated with bacterial metabolism, extending well beyond folate metabolism. Since we intended show the metabolites with their names, it was impossible to show all 800 plus metabolites. As an alternative we provided the abundance data in the supplementary table which enlists all 886 metabolites detected in our mass spectrometric analysis. Further to avoid any biased representation of the perturbed global metabolome we also catalogued metabolites comprising carbohydrate metabolism along with cofactors and peptides in the representative heatmap (Figure 2A).

• The differences in metabolite abundances observed at 5h and 12h (Figure 2A) could be due to the differences in growth rates as the presence of the drug slows down growth and metabolic processes, rather than being a direct consequence of the drug mode of action.

Response:

We thank the reviewer for raising this point. To avoid any confusion in interpreting the timepoints we changed the time-labels in the revised draft. It was ensured that same cellular mass was used for harvesting cells and processing them for further downstream steps before carrying out mass spectrometry.

• As the carbon source is only provided at 0.8 g/L, it is expected that the non-treated cells will rapidly enter stationary phase (at 12h, and probably even at 5h depending on the density inoculated). Is it thus pertinent to compare the metabolome of cells that reached stationary phase with metabolome of cells that are still growing the differences in nucleotide biosynthesis. It should be more relevant to perform metabolomics after 1 or 2 hours of treatment, or to grow the cells with a highest amount of C-source to have a longer exponential growth phase.? Considering this, only the metabolome at 30 min appears to be relevant. This could notably explain

Response:

We thank the reviewer for this point. To probe into the comparative global metabolome upon CD15-3 induced stress we intended to capture metabolic perturbation at three major points of bacterial growth viz. the early log phase (~30 minutes), mid exponential phase (~5 hours) and the point when the cells enter stationary phase (~12 hours). These time points were selected based on the growth profile of the CD15-3 treated cells. These three major time points helped us to capture stress-induced metabolic perturbation at three discrete metabolic stages viz. from the phase of metabolic adaptation to the end of active bacterial growth. We used an extremely low inoculum load to start our culture and made sure all the culture sets have exactly same cell mass. Further our growth experiments showed that CD15-3 stressed cells do not enter stationary phase at 5 hours, rather they enter stationary phase after around 12 hours of growth.

• In the 3rd paragraph of the section, the assumption that metabolites displaying the most delayed recovery are the most impacted upon the treatment could be incorrect. Indeed, it also strongly depends on the flux of matter generating the metabolite. It is expected that a central metabolite associated with high matter fluxes, such as pyruvate, will have a fastest recovery than an intermediate metabolite involved in vitamin biosynthesis for example.

Response:

The reviewer is correct that recovery time could be sensitive to basal uptake rates of the metabolites. We note that among the metabolites greatly perturbed, there were metabolites in central metabolic pathways that recovered relatively fast (pyruvate, AMP), and those in peripheral metabolic pathways that recovered relatively slowly (thymidine, AICAR), as anticipated by the reviewer. However, we note that there are also relatively slow recovering metabolites in central metabolism (citrate) and fast recovering metabolites in peripheral pathways (NAG), and thus there seem to be differences in recovery dynamics that supersede pathway structure alone.

This is a very interesting line of inquiry however, and we have now revised the paragraph on recovery to better motivate the experiment as well as to mention these complications in analysis (*Lines 162-171*)

• The need to perform the recovery assay (Fig. 2C and 2D and 3rd paragraph of the section) is unclear. Is it an essential part of the main workflow proposed? It doesn't seem so, as it is not directly used in the next part. The authors should emphasize its use in the workflow, or alternatively remove the section.

Response:

We apologize for this being unclear in the original version. The idea behind the recovery experiments is that the pathways that recover slowly may be the most impacted or be the most problematic to growth. We were investigating whether there could be metabolites that can rapidly alter their levels without serious physiological effect while inability of other metabolites to adjust could indicate underlying problems in maintaining growth homeostasis. As mentioned in the above comment, we observed some metabolites did respond more slowly than expected, and some of these such as thymidine ended up being successful supplements for rescuing growth.

As mentioned above, we have now revised the recovery paragraph to better motivate these experiments and the intended interpretation of the results. *Lines 162-171*

Section "Machine learning reveals antibiotic mechanism-specific perturbations"

• In the section, the authors show with a LR model that their metabolome profile fits well with the metabolome profile of an antifolate drug. This was strongly expected as the drug was originally designed to interact with dihydrofolate reductase (Zhang et al., 2021). Thus, the quality and relevance of the LR model should be better proven to validate its use in the workflow. For example, the authors could perform similar metabolomics assay on different drugs (with different targets), and test whether the model predicts adequately their class of mechanism.

Response:

The reviewer raises an important point. We did test other drugs from the Zampieri study, but found the performance was low for non-metabolic drug classes. This diversity was noted by Zampieri et. al. as well in their original study. We found that predictions did not work for other mechanisms in the Sauer dataset, which we believe is most likely due to a non-metabolic basis of inhibition therefore diverse metabolic side effects. For this reason, we believe that the workflow (or any novel target-finding workflow based on metabolomics data) is most likely to be successful for drugs with metabolic (but potentially multiple) targets. We had noted this limitation in our original manuscript, so we hope this explanation makes sense to the reviewer why we could not expand this section of the work without extensive data generation.

• How would the proposed workflow work if the drugs have a mode of action different from the five classical mechanisms proposed? As the research for novel antibiotics aims at finding alternatives to classical mode of actions, using an approach only based on data on the classical mode of actions seems irrelevant.

Response:

This is an excellent question. As the reviewer suggests, this work is primarily suited for antimetabolites, i.e., drugs targeting metabolic enzymes, which are mostly outside the classical mechanisms of antibiotic growth inhibition. However, the machine learning workflow based on comparing the novel drug metabolomics response to the response of other characterized drugs is intended to separate generic growth inhibition effects from mechanism-specific effects from drug-specific effects. Thus, it is not required that the drug be related to any of the five main drug classes.

This is a very interesting and important point and we have added a comment related to this in the machine learning section. *Lines 200-203*

Section "Metabolic modeling predicts patterns in growth rescue experiments for candidate pathway inhibitions"

• The choice of the nutrients used for supplementations and for the subsequent parts of the study is unclear and seems to be arbitrary. IMP was selected from the RL model (Fig 2E), but: i) uacgam, argsuc, cbasp, cys, mnl1p and g3pi were also identified by the model. Using a pragmatic workflow, these should also be included in the supplementation. ii) no clear justification is brought for the inclusion of glycine, serine, thymidine, orotate, uridine is the supplementation assay. As already commented, these arbitrary choices seem to show bias in the study due to a previous idea of the mode of action, rather than a rigorous workflow.

One justification given is that the authors selected metabolites close to folate pathway. This seems a contestable as a drug could target similarly enzymes from very distinct pathways. Furthermore, metabolism is organized as a highly interconnected network rather than by independent pathways. Thus, selecting which metabolites are close or not to a given pathway is difficult to assess pragmatically without a graph-based computational approach.

Response:

We thank the reviewer for pointing out that the rationale behind choice of supplements was unclear. Supplemented metabolites were chosen based on consideration of 1) results from both raw metabolomics and resulting machine learning, which indicated an antifolate-like signature as well as identifying other specifically perturbed metabolites 2) annotation of transporters that would enable the uptake of the supplemented metabolite in E. coli, and 3) commercial availability of the metabolite. We note that the folate signature from machine learning (Figure 3E) was not intended to directly motivate the entire supplement list; instead, we utilized it to inform antifolate-related metabolic perturbations and possibly distinguish those metabolites from CD15-3-specific metabolic perturbations. We note that IMP was shared as a target between metabolomics data alone and the folate signature, and it was a supplement that successfuly rescued growth, which helped to further support that the CD15-3 growth inhibition was occurring via the folate pathway.

To discuss the specific examples highlighted by the reviewer, thymidine was a highly perturbed metabolite in the metabolomics data that showed slow recovery experimentally, and transporters for thymidine exist in E. coli, and therefore it was chosen as a supplementation candidate despite not appearing among top scoring metabolites in the folate signature from machine learning. We believe that thymidine may be a CD15-3-specific perturbation that is not shared among the broader pool of anti-folate drugs, and therefore it is still of interest in a workflow attempting to find the target of CD15-3.

By comparison, mannitol-1-phosphate was a high scoring metabolite in the folate signature from machine learning, but it does not have an annotated transporter and therefore it would be unclear whether lack of growth rescue would be due to lack of a physiological effect of the supplemented metabolite or merely the inability of the cell to take up the metabolite efficiently. While related metabolites (such as mannitol) could be substituted when a transporter or commercial metabolite is not available, we believed that this would complicate the resulting analysis.

To address this comment, we now included a supplementary table listing transporter availability, which alongside the other data already provided, we hope makes the rationale to use these compounds as media supplements clear. We also expanded the section motivating the supplements in the text. *Lines 258-262*

• 2nd paragraph of the section: Even though the metabolite tested cannot be a sole carbon source, their complementation on a medium with glucose as C-source could boost the growth and induce a higher growth rate. A growth assay of nutrient supplementation on glucose medium should be performed to determine this potential growth rate increase and taken into account before drawing conclusion from Figure 4A. Indeed, the fact that some nutrients increase the growth of treated bacteria could be theoretically independent of the drug mode of action.

Response:

We thank the reviewer for raising this concern. In our earlier version of the manuscript, we reported that the select metabolites used for supplementation experiment do not alter the growth rate of the growing cells (Supplementary Figure: 2A). In the current revised version of the draft, we have further shown that these metabolites individually upon supplementation do not affect the lag time of the growing cells (Supplementary Figure: 2D).

• The fact that NAG and serine prolonged the lag phase could be related to an effect on the pH of the medium. The authors should check if adding them change importantly the pH.

Response:

We thank the reviewer for this interesting suggestion. We carried out our growth experiments and kept tracking the pH of the media under all the conditions where metabolic supplements were added (one metabolite at a time). We did not observe metabolite supplementation induced pH change. We discussed the results in the paper (*Lines 273-275*) and included the new results as a supplementary figure (Supplementary Figure:2C).

• The authors claim that they both tested in silico the effect of nutrient supplementation on i) reaction inhibition ii) cofactor depletion. However, the authors only show one of these results on Figure 3F, 3G, 3H: only one in silico growth is displayed by nutrient. It is unclear which one is depicted. Thus, the claim that “the experimental growth rescue pattern was most consistent with a folate cofactor drain mechanism”. The authors should rework the way they present their metabolic modeling results on this section.

Response:

We thank the reviewer for pointing out this ambiguity. We have now changed the figure caption to make it clear the metrics used for growth rescue in each simulation. *Lines 242-248*

We note that the full code is made available that details the constraints and objectives used, which we hope enables transparency.

• The authors explain that first, the metabolic models were not able to correctly predict growth rescue with nutrient complementation. They performed several manual modifications of the metabolic model, justified by expression data or spontaneous reactions, to have a better consistency with experimental

results. The need to manually modify the models to have a fit with experimental data is questionable and makes the workflow less straightforward than what was expected. This manual modification of the metabolic model seems to have only been done around folate metabolism. It would be preferable to have an automatic way to integrate expression data and spontaneous reactions as mathematical constraints, and then perform the computational analysis on the resulting metabolic model, rather than manually (and potentially with bias) modify what does not fit the authors expectations.

Response:

The reviewer is correct that the implementation of the nutrient supplementation simulation involved pathway-specific modifications to the model. These changes reflected the expression state of the cell under the condition of interest. Methods have been developed to automate expression constraints, such as the GIMME algorithm (Becker and Palsson, 2008). Similarly, removing spontaneous reactions globally in an automated fashion is currently possible, as these reactions are annotated with the artificial gene ‘s0001’, and thus all such reactions can be removed prior to simulation. However, we did not use these methods in our case study because we were focused on a manageable number of nutrients and manual intervention seemed reasonably simple. Oftentimes automated methods have a number of error cases that require extensive debugging regardless, so we believe that manual fixes to model structure around particular known nutrients is a valid alternate approach. Changes to the global metabolic network are not appropriate in this case because changes in gene expression will be a condition-specific set of constraints. We agree that manual interventions can be subject to bias. However, we hope that the considerations we used here (lack of expression and reaction spontaneity) are transparent and transferable, and therefore could be considered relatively bias-free.

We now describe alternative approaches in the supplementation section of the Results. (*Lines 342-345*).

Section “Structural analysis of possible alternate binding targets”

• It is unclear how the authors would have performed the analysis without their a priori knowledge that the drug was designed to target DHFR, as the selection is based on the assumption that the drug should target a protein structurally close to DHFR.

Response:

The reviewer raises an excellent point that we did not originally explain well in the manuscript. In our case study, we already knew folate was likely relevant, DHFR was an intended CD15-3 target, and therefore first and foremost we focused on proteins within the folate pathway and neighboring pathways. If we did not know that DHFR was a target already, we would have to prioritize enzymes based on likelihood of docking and substrate similarity. This was not described in the manuscript, but it is critical to mention. We have now included an explanation of the intended de novo workflow in the Discussion. *Lines 597-602*.

Material and Methods

There is a lack of consistency in the Material and Methods section. The authors should start by giving the species and strain name of the bacteria used (Escherichia coli BW25113) instead of using expressions such as “Bacterial cultures”, “WT cells” “WT BW25113”etc which are confusing.

Also, please clearly refer at the beginning that: M9 medium with glucose at 0.8 g/L was used during all the experiments (unless otherwise stated), as in some part only “M9 medium” is written, which is misleading. Also, the authors should mention clearly which drug concentration(s) where used, and clearly specify if it was not the same in all the results presented, and why.
Same consistency should also be used in figure descriptions and result section.

Response:

We thank the reviewers for raising these concerns. In the current revised version of the manuscript, we have addressed the issues mentioned and made these nomenclatures uniform.

Minor comments

• Figure 2B and 2C. The Y axis is the same: “Metabolite abundance fold change CD15-3 pre-exposed vs naïve (log scale)” and makes it difficult to understand the difference between the two figures.

Response:

We thank the reviewer for pointing this out. We rectified the axis labels for Figure 2B and 2C.

• Please improve the consistency in the spelling of “modeling”, that is either spelled modelling or modeling depending on the section.

Response:

Thanks for pointing this out –Fixed.

<u>Reviewer #3 (Remarks to the Author):</u>

This manuscript guides you through a workflow that uses metabolomics data in combination with machine learning techniques, metabolic models, and structural analyses to suggest priority candidates for drug target studies.
The workflow was presented rather well at the beginning of the manuscript (Figure 1 and description). The Jupyter notebook that came attached to the manuscript was also highly appreciated. There are however some points that are not entirely clear to me.

1) Firstly, I would like to point out a few things that require immediate rectification prior to publication. The figures in the manuscript (e.g., supplementary Figure 4) are of subpar quality and are at many points hard to read. The equations do not carry any equation numbers, making them rather difficult to refer to. Additionally, some equations seem to swap around indices (i.e., subscripts or superscripts for x0). Some equations also have symbols that are not properly rendered, at least in the manuscript that I received. I attached some screenshots that might help.

<u>Response:</u>

We thank the reviewer for pointing out these two issues. In the revised version of the manuscript, we replaced the supplementary figure 4 with a figure with better resolution. We also made changes in the notations used in the equations.

2) Regarding the machine learning approach presented in the paragraphs: "Machine learning reveals antibiotic mechanism-specific perturbations" and "material and methods". If I understood correctly, a "previously published dataset on the metabolomic response of different antibiotics" was used as a starting point for machine learning to identify specific perturbations of the drug. How is this initial data pool selected (especially for a potential application of this workflow in the future)?

<u>Response:</u>

The external drug-treated metabolomics dataset (Zampieri et. al.) was used to develop a background of metabolic perturbations to separate drug-specific perturbations from mechanism-specific perturbations from generic growth inhibition-related perturbations. When selecting a valid dataset to use as a background, the most important feature is to have a large dataset from a single study, to minimize batch effects and other technical artifacts than can arise when combining datasets. The second requirement is to have within the panel of drugs, those with defined mechanisms as opposed to uncharacterized compounds. This enables us to perform supervised machine learning to discover mechanism-specific perturbations. There are a few such datasets published recently (both by Zampieri et. al.) that hopefully will continue to grow into a background of antibiotic-induced cellular responses that can be used to anticipate the effects of novel compounds, as has been possible with gene expression data using the Broad Institute's Connectivity Map, for example.

In response to the reviewer's inquiry, we now mention these points to the Discussion section. *Lines 568-570.*

3) There are many regression models available to perform data classification. Why was a multiclass logistic regression model chosen in this study? Does it have any particular advantages?

<u>Response</u>

Logistic regression is one of the simplest classification machine learning approaches, as it uses a linear combination of features to predict class. Because it is simple, it is less vulnerable to overfitting when we have a small dataset, as is the case in our study. We found that other methods showed worse performance in terms of test accuracy across the drug classes in the Zampieri dataset. Unfortunately, we are in a relatively data-poor situation due to only having metabolomics data for a set of ~10 compounds, compared to hundreds of measured metabolites, so we were not in a position to utilize more sophisticated ML that typically requires a deeper dataset. Hopefully this work, and those of the authors who generated the high-throughput metabolomics data (Zampieri et. al), will be inspirational to the community by demonstrating the value of this data for drug target finding.

4) It is also not completely clear to me how the performance of the machine learning model was evaluated. In the manuscript, it was written that default parameters have been used. Following this, the authors carried out a leave-one-out cross-validation. How exactly would the performance be evaluated, if the parameters are kept the same?

Response/Action: (Daniel)

Performance was evaluated over different train-test splits during cross-validation, averaging performance over separate splits. Default hyperparameters for regularization were used when generating the logistic regression model, resulting in models with different parameters (e.g. linear coefficients). We have adjusted the wording in the methods to better explain this. *Lines 685-687*.

5) I would also like to ask a quick question about Figure 2 B. What exactly has been plotted? Fold-change differences between pre-exposed and naive? I have also been told many times that timepoints in metabolomics data can be a quite delicate subject. How have the time intervals been selected?

Response:

We thank the reviewer for raising this point. There was an inadvertent error in the axis label of Figure 2B. We have rectified that in the revised manuscript. It was ensured that same cellular mass is used for harvesting cells and processing them for further downstream steps before carrying mass spectrometry.

References:

Becker, S.A., and Palsson, B.O. (2008). Context-specific metabolic networks are consistent with experiments. PLoS computational biology *4*, e1000082.
Bhattacharyya, S., Bershtein, S., Yan, J., Argun, T., Gilson, A.I., Trauger, S.A., and Shakhnovich, E.I. (2016). Transient protein-protein interactions perturb E. coli metabolome and cause gene dosage toxicity. elife *5*, e20309.
Chhabra, S., Dolezal, O., Collins, B.M., Newman, J., Simpson, J.S., Macreadie, I.G., Fernley, R., Peat, T.S., and Swarbrick, J.D. (2012). Structure of S. aureus HPPK and the discovery of a new substrate site inhibitor. PloS one *7*, e29444.
Marimuthu, P., Singaravelu, K., and Namasivayam, V. (2017). Probing the binding mechanism of mercaptoguanine derivatives as inhibitors of HPPK by docking and molecular dynamics simulations. Journal of Biomolecular Structure and Dynamics *35*, 3507-3521.
Shi, G., Blaszczyk, J., Ji, X., and Yan, H. (2001). Bisubstrate analogue inhibitors of 6-hydroxymethyl-7, 8-dihydropterin pyrophosphokinase: synthesis and biochemical and crystallographic studies. Journal of medicinal chemistry *44*, 1364-1371.
Zampieri, M., Szappanos, B., Buchieri, M.V., Trauner, A., Piazza, I., Picotti, P., Gagneux, S., Borrell, S., Gicquel, B., and Lelievre, J. (2018). High-throughput metabolomic analysis predicts mode of action of uncharacterized antimicrobial compounds. Science Translational Medicine *10*, eaal3973.
Zhang, Y., Chowdhury, S., Rodrigues, J.V., and Shakhnovich, E. (2021). Development of antibacterial compounds that constrain evolutionary pathways to resistance. Elife *10*, e64518.
Zhou, W., Ramos, E., Zhu, X., Fisher, P.M., Kidane, M.E., Vanderloop, B.H., Thomas, C.D., Yan, J., Singha, U., and Chaudhuri, M. (2019). Steroidal antibiotics are antimetabolites of Acanthamoeba steroidogenesis with phylogenetic implications. Journal of lipid research *60*, 981-994.

REVIEWER COMMENTS

Reviewer #1 (Remarks to the Author):

Having studied the revised manuscript, I find that the authors have satisfactorily addressed all queries raised by me and other reviewers. This is an exciting study with a lot of potential and I recommend that it be accepted for publication without further revision.

Reviewer #2 (Remarks to the Author):

I thank the authors for their detailed response to the concerns I raised.

Section An integrated metabolomics-guided framework to identify intracellular drug targets

• I noticed the change in Figure 2A: 30 min, 5h, 12h modified into early log, mid exponential, late log. According to the authors response, it seems to be done according to the phases observed with CD15-3 treated cells. It still does not answer to my concern that the non-treated cells could be at a different growth phase if CD15-3 induces a significant effect on growth rate. A growth curve (or even some OD measurements the authors should have) of WT and WT + CD15-3 cells at the sampling time would help answer that.

• Also, I have read on the authors response that cells were harvested and processed at the same cellular mass. However, even if the same cellular mass is used, it does not remove the issue that metabolites could have contrasted abundance depending on the growth phase. We expect a strong biosynthetic activity at the beginning of exponential phase versus a poor biosynthetic activity at the end of the exponential phase.

Section Machine learning reveals antibiotic mechanism-specific perturbations

Authors Reponse: The reviewer raises an important point. We did test other drugs from the Zampieri study, but found the performance was low for non-metabolic drug classes. This diversity was noted by Zampieri et. al. as well in their original study. We found that predictions did not work for other

mechanisms in the Sauer dataset, which we believe is most likely due to a non-metabolic basis of inhibition therefore diverse metabolic side effects. For this reason, we believe that the workflow (or any novel target-finding workflow based on metabolomics data) is most likely to be successful for drugs with metabolic (but potentially multiple) targets. We had noted this limitation in our original manuscript, so we hope this explanation makes sense to the reviewer why we could not expand this section of the work without extensive data generation.

• It is concerning that the revised paper still claims providing a “systemic” workflow and that “machine learning reveals antibiotic mechanism-specific perturbations” if machine learning only works with the molecule from the authors lab (CD15-3) and the model does not work with other molecule.

Section Metabolic modeling predicts patterns in growth rescue experiments for candidate pathway inhibitions

• I thank the authors for explaining how they selected metabolites for supplementation. I understand they removed metabolites with no associated transporters or no/limited commercial availability. However, for the other metabolites, the authors should provide a pragmatic pipeline to explain how metabolites are selected based on perturbation of the abundance (maybe with a fold-change threshold?). Also, once again, I am not sure to follow how this workflow would be used if the molecule was not known to target DHFR. And also, the authors do not answer to my concern that “proximity” in an interconnected metabolic network is a complex question and “close metabolites” should be found by a pragmatic assessment rather than by an arbitrary claim that the metabolites are close.

Global remark

I asked at each step (machine learning, metabolic modeling, structural modeling) how the workflow should work without a priori on the drug mode of action. I appreciate that the authors acknowledged this is a limitation of their study. However, I find the title of the paper “empowering systems-guided drug target” as well as the abstract mentioning “a systems-guided hierarchic workflow” misleading as the authors did not manage to show their workflow could be applied to another molecule with no a priori on it.

Reviewer #3 (Remarks to the Author):

Most of my questions about the manuscript, that guides through a workflow that uses metabolomics data in combination with machine learning techniques, has been answered.

point 2 reviewer 3)

I appreciate the new explanations in the paragraph about machine learning approach, I think it is clearer now.

point 3 reviewer 3)

About this point I would say that in general I am always a bit skeptical when it comes to dataset with poor-data situations (as called by the author), simply because machine learning by definition requires good datasets. However, I also understand that the authors tried to do their best with the data available.

I still have something to say about the format of the equations at the end. Some mathematical symbols are still not readable when I download the PDF version (example line 804). Please make sure everything is correct and readable.

RESPONSE TO REVIEWER'S COMMENTS

Manuscript Title: Empowering systems-guided drug off-target discovery with metabolic and structural analysis

Manuscript ID: NCOMMS-22-28234

We sincerely thank all reviewers and the editor for their keen interest in our work and for the valuable comments in revision rounds and suggestions to improve the manuscript. We thank reviewer 1 and 3 for approving the work and acknowledging the revisions made.

We have revised the article based on the remaining comments and suggestions from reviewer 2 and 3. The reviewers mentioned important points and we appreciate the opportunity to clarify these points for the improvement of our manuscript. Necessary changes as per the comments have been made in the manuscript at appropriate places (underlined and marked with red).

Reviewer Comments:

Reviewer #1 (Remarks to the Author):

Having studied the revised manuscript, I find that the authors have satisfactorily addressed all queries raised by me and other reviewers. This is an exciting study with a lot of potential and I recommend that it be accepted for publication without further revision.

Response:

We thank the reviewer for appreciating the revisions made and accepting the manuscript for publication.

Reviewer #2 (Remarks to the Author):

I thank the authors for their detailed response to the concerns I raised.
Section An integrated metabolomics-guided framework to identify intracellular drug targets

• I noticed the change in Figure 2A: 30 min, 5h, 12h modified into early log, mid exponential, late log. According to the authors response, it seems to be done according to the phases observed with CD15-3 treated cells. It still does not answer to my concern that the non-treated cells could be at a different growth phase if CD15-3 induces a significant effect on growth rate. A growth curve (or even some OD measurements the authors should have) of WT and WT + CD15-3 cells at the sampling time would help answer that.

Response:

We thank the reviewer for pointing this out. For better clarity we have incorporated the comparative growth profiles of treated and untreated (control) cells and provided additional explanations in the main text as well as figure legend (Supplementary Figure 1). In the revised text while mentioning the time points for metabolomic analysis we have specifically mentioned "cells at their respective" early log phase, mid exponential phase, and late log phase. We acknowledge that use of time point values in the earlier text was leading to confusion in interpreting the respective growth phases for untreated and treated cells. Also, throughout the text to avoid confusion associated with time points we have replaced earlier used time point numbers with the corresponding phases of growth.

• Also, I have read on the authors response that cells were harvested and processed at the same cellular mass. However, even if the same cellular mass is used, it does not remove the issue that metabolites could have contrasted abundance depending on the growth phase. We expect a strong biosynthetic activity at the beginning of exponential phase versus a poor biosynthetic activity at the end of the exponential phase.

Response:

We thank the reviewer for this point. However as mentioned earlier we harvested the cells at their same respective growth phase. Cells in the same growth phase are likely to have same extent of metabolic adaptation with the ambient culture medium. The differences in the metabolite levels which showed up in the comparative metabolome for the treated versus untreated cells at the same phase of growth was used for the analyses to identify the drug induced hotspots of metabolic perturbations.

Section Machine learning reveals antibiotic mechanism-specific perturbations.

Authors Response: The reviewer raises an important point. We did test other drugs from the Zampieri study, but found the performance was low for non-metabolic drug classes. This diversity was noted by Zampieri et. al. as well in their original study. We found that predictions did not work for other mechanisms in the Sauer dataset, which we believe is most likely due to a non-metabolic basis of inhibition therefore diverse metabolic side effects. For this reason, we believe that the workflow (or any novel target-finding workflow

based on metabolomics data) is most likely to be successful for drugs with metabolic (but potentially multiple) targets. We had noted this limitation in our original manuscript, so we hope this explanation makes sense to the reviewer why we could not expand this section of the work without extensive data generation.

• It is concerning that the revised paper still claims providing a "systemic" workflow and that "machine learning reveals antibiotic mechanism-specific perturbations" if machine learning only works with the molecule from the authors lab (CD15-3) and the model does not work with other molecule.

Response:

We understand the reviewer's point and agree that whether the workflow is generalizable to find the targets of general compounds is still undetermined. We still argue that the use of the workflow in discovering off-targets of metabolic enzyme inhibitors, such as CD15-3, is validated by this study. We consider discovering and validating off-targets of multiple other compounds to prove generality of the workflow to be beyond the reasonable scope of this study, requiring additional metabolomics, supplementation, and overexpression experiments.

To address the reviewer's continued concerns, we have made two changes to the description of the work. First, we have specified in several locations that the workflow is developed for the case of identifying off-targets of metabolic inhibitors like CD15-3. Second, we have now removed mention of CD15-3 being a 'case study' for a general target finding workflow in many places in the text, and have adjusted the description of the workflow to try to make it clear that the workflow is utilized to discover the target of CD15-3 in particular. Please see the modified title and lines 19-25, 50-67, 88-90, 104-105, 616, and 637 for the relevant text.

We hope that these changes better specify the use cases for which we have evidence that the workflow is effective.

Section Metabolic modeling predicts patterns in growth rescue experiments for candidate pathway inhibitions

• I thank the authors for explaining how they selected metabolites for supplementation. I understand they removed metabolites with no associated transporters or no/limited commercial availability. However, for the other metabolites, the authors should provide a pragmatic pipeline to explain how metabolites are selected based on perturbation of the abundance (maybe with a fold-change threshold?).

Response:

We have now attempted to further refine the description of the workflow to choose supplements. We note that we did not use quantitative criteria, e.g. recommending a specific fold-change threshold, given the complications of interpreting metabolomics data from compounds from diverse pathways. It is unclear to us, for example, whether a 3-fold change to serine concentration would be functionally more impactful than a 2-fold change to ATP concentration. Instead, we took the approach of trying to achieve broad coverage of relevant-seeming compounds, and trying to extract a functional inference from the subsequence trends in growth rescue experiments. We further note that there is not expected to be a unique set of useful

supplements, as neighboring compounds would often be expected to provide similar information, provided that transporter and other criteria are met.

Please see lines 256-271 for these changes.

Also, once again, I am not sure to follow how this workflow would be used if the molecule was not known to target DHFR.

Response:

We agree that the workflow is aided by previously establishing a known target of the drug, something that we acknowledge in the text. We acknowledge that the workflow is better described as an off-target finding workflow rather than an *a priori* generic target finding workflow, and we've modified the title accordingly. That said, with no idea of where the initial target could be, we could still recommend a workflow consisting of 1) metabolomics with comparison to previously characterized antibiotic compounds, 2) supplementation to probe growth inhibited pathways, and 3) overexpression of candidate targets to attempt a growth rescue. Structural analysis could still be deployed to computationally assess the possibility of multi-targeting of the drug. We have added a mention of these aspects of the workflow that could be applicable to generic target finding to the Discussion (Line 613 to 622).

And also, the authors do not answer to my concern that "proximity" in an interconnected metabolic network is a complex question and "close metabolites" should be found by a pragmatic assessment rather than by an arbitrary claim that the metabolites are close.

Response:

We apologize for missing this comment previously. We agree that closeness in a metabolic network is a complex question, as cofactors, protons, and other small molecules like water and CO_2 can increase the connectivity of a network in ways that do not follow traditional pathway definitions nor intuition of what should constitute a meaningful network connection. However, these issues can be handled reasonably well either quantitatively or qualitatively. Quantitatively, metabolites beyond a certain connectivity can be excluded when calculating distances between metabolites or reactions, the threshold for which being established by examining the distribution of metabolite connectivities. In the case of this workflow, we utilized a qualitative approach based on canonical pathway definitions established by manual curators, therefore matching a textbook understanding of proximity between metabolites. We now include this definition of proximity in the section where supplementation is mentioned (Line 254 to 268).

We hope that these additions to the manuscript text make the choice of supplements now clear and establish a workflow that can be followed in new cases.

Global remark

I asked at each step (machine learning, metabolic modeling, structural modeling) how the workflow should work without a priori on the drug mode of action. I appreciate that the authors acknowledged this is a limitation of their study. However, I find the title of the paper "empowering systems-guided drug target" as well as the abstract mentioning "a systems-guided hierarchic workflow" misleading as the

authors did not manage to show their workflow could be applied to another molecule with no a priori on it.

Response:

We thank the reviewer for this comment and understand the perspective. In the revised version of the manuscript, as per the suggestion we have changed relevant sections in the abstract and main text of the manuscript along with slightly modifying the title to avoid any confusion. These changes mostly consistent of avoiding the mention of this workflow as a general drug target finding workflow and instead specifying the use case for identification of off-target activity as presented in this work.

Reviewer #3 (Remarks to the Author):

Most of my questions about the manuscript, that guides through a workflow that uses metabolomics data in combination with machine learning techniques, has been answered.

point 2 reviewer 3)

I appreciate the new explanations in the paragraph about machine learning approach, I think it is clearer now.

point 3 reviewer 3)
About this point I would say that in general I am always a bit skeptical when it comes to dataset with poor-data situations (as called by the author), simply because machine learning by definition requires good datasets. However, I also understand that the authors tried to do their best with the data available.
I still have something to say about the format of the equations at the end. Some mathematical symbols are still not readable when I download the PDF version (example line 804). Please make sure everything is correct and readable.

Response:

We thank the reviewer for appreciating the revisions made and pointing out the issue with mathematical symbols in the equation. In the revised version of the manuscript, we have rectified the issue.
